# Ultrastrong Host–Guest Recognition Driven Chiral Supramolecular Polymers with Circularly Polarized Luminescence in Water

**DOI:** 10.3390/polym18172040

**Published:** 2026-08-22

**Authors:** Ya-Ping Chen, Si-Dan Guo, Jinlei Zhou, Xiaoyu Luo, Kang Cai

**Affiliations:** State Key Laboratory of Elemento-Organic Chemistry, Frontiers Science Center for New Organic Matter, College of Chemistry, Nankai University, Tianjin 300071, China; 2120241198@mail.nankai.edu.cn (Y.-P.C.); 1120220495@mail.nankai.edu.cn (S.-D.G.); zjl201226@163.com (J.Z.); 1120250686@mail.nankai.edu.cn (X.L.)

**Keywords:** ultrastrong host–guest recognition, supramolecular polymer, chirality, circularly polarized luminescence

## Abstract

Chiral supramolecular polymers offer a versatile platform for constructing dynamic functional materials, yet their development in aqueous media remains challenging due to weak host–guest interactions and limited chiral macrocyclic systems. Herein, we report a water-soluble chiral luminescent supramolecular polymer assembled from a pair of chiral macrocycle ((*R*)**-**/(*S*)**-C[4]B**) and an achiral bis-thiazole orange guest. The ultrahigh binding affinity between the host and guest enables stable one-dimensional polymerization in water. Efficient chirality transfer from the macrocyclic host to the achiral luminophore gives rise to pronounced circular dichroism (CD) and circularly polarized luminescence (CPL). The resulting polymer exhibits red emission (625 nm) with a luminescence dissymmetry factor of 8.5 × 10^−3^. This work provides an effective strategy for the development of aqueous CPL-active supramolecular polymers through ultrastrong host–guest recognition.

## 1. Introduction

Supramolecular polymers, constructed from molecular building blocks through reversible noncovalent interactions such as hydrogen bonding, π-π stacking, metal coordination, and host–guest recognition, have attracted sustained attention over the past decades [1,2,3]. Compared with covalent polymers, they offer unique advantages, including dynamic reversibility, stimuli responsiveness, adaptive reconfiguration, and self-healing behavior [4,5,6,7]. These features enable the construction of soft, programmable, and stimuli-responsive materials that are difficult to achieve in traditional polymer systems, making supramolecular polymers a long-standing research focus in the field of supramolecular chemistry. The introduction of chirality further expands the structural and functional dimensions of supramolecular polymers [8,9]. On one hand, chiral supramolecular assemblies provide valuable model systems for understanding biological chirality transfer and amplification processes [10,11,12,13]. On the other hand, they enable a broad range of emerging applications, including chiral sensing, bioimaging, and chiral optoelectronic devices [14,15,16,17]. In particular, CPL-active materials, which integrate luminescence and chiroptical information, have attracted significant interest due to their potential in three-dimensional displays, optical data storage, and advanced photonic technologies [17,18,19]. Therefore, the development of efficient strategies to construct chiral supramolecular polymers with controllable structure and strong chiroptical responses remains an important and active research direction.

Among various noncovalent interactions, host–guest recognition represents a particularly powerful and versatile platform for constructing supramolecular polymers. The rapid development of macrocyclic host chemistry has provided a diverse library of binding motifs, while guest molecules can be readily tailored to incorporate functionalities such as fluorescence, redox activity, drug activity, or stimuli-responsiveness [20,21,22,23,24,25,26,27]. Importantly, the use of water-soluble macrocycles such as cyclodextrins [28,29,30,31,32] and cucurbiturils [33,34,35,36,37,38,39,40,41,42,43,44,45,46] enables the construction of biocompatible supramolecular polymer systems in aqueous media, offering unique advantages over other interaction modes such as hydrogen bonding or π-π stacking. However, except for a few highly affine systems such as cucurbituril-based complexes [47], most host–guest interactions exhibit binding constants below 10^6^ M^−1^, which is often insufficient to maintain stable polymeric assemblies against dilution. In addition, the scarcity of robust chiral macrocyclic hosts with strong and directional recognition ability further limits the development of chiral host–guest supramolecular polymers. Taken together, we believe that the construction of CPL-active supramolecular assemblies based on host–guest interactions in aqueous media mainly faces four challenges: (1) how to achieve sufficiently strong and directional host–guest interactions in aqueous media to support supramolecular assembly; (2) whether effective chirality transfer can be achieved upon host–guest complexation; (3) whether the assembled system can retain sufficient luminescence after host–guest recognition; and (4) CPL-active materials capable of emitting in the red to near-infrared region remain relatively scarce.

**Scheme 1:**
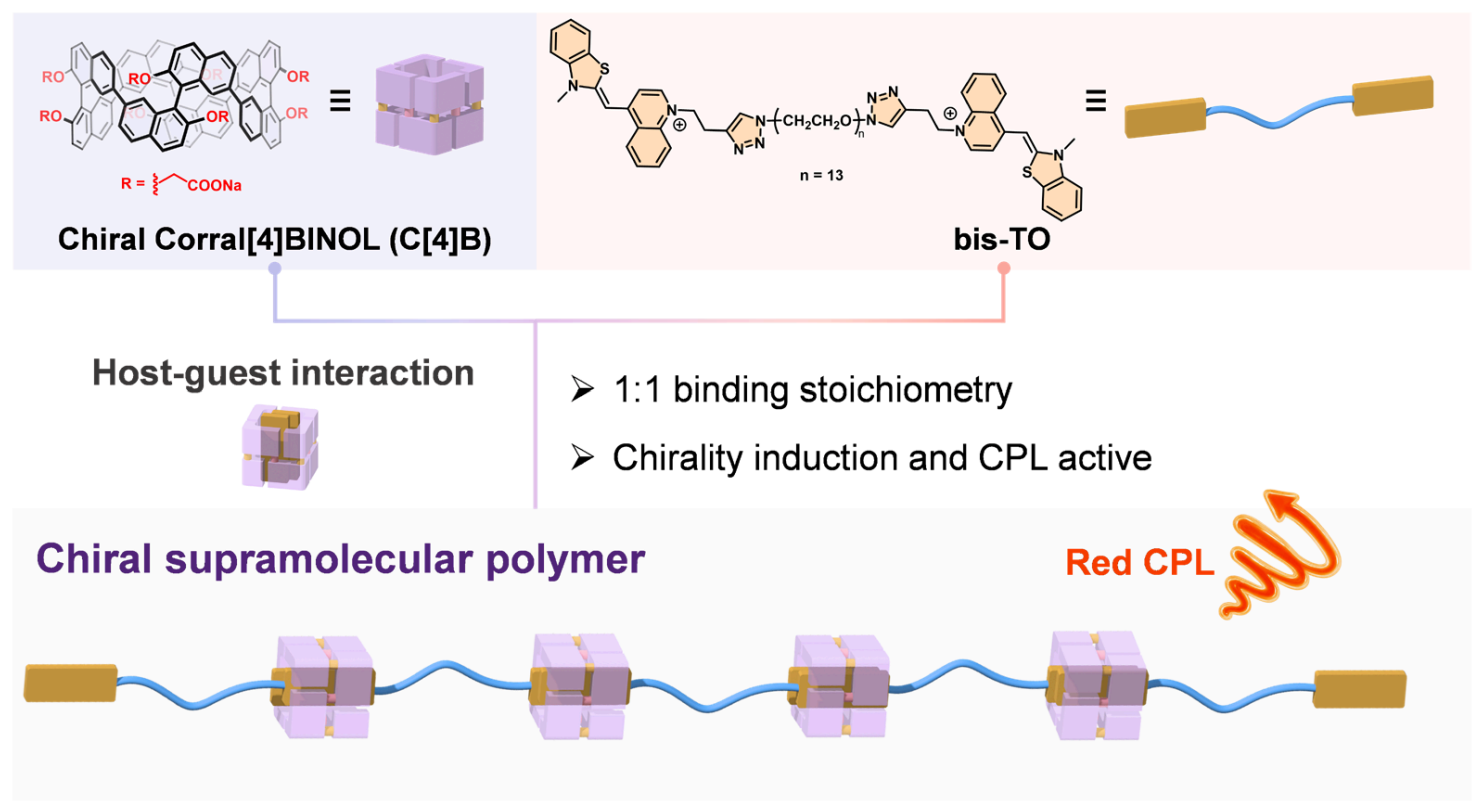
Schematic illustration of the supramolecular polymerization of **bis-TO**@**C[4]B** based on host–guest interaction.

Herein, we report a water-soluble chiral luminescent supramolecular polymer constructed from a chiral Corral [4]BINOL macrocycle ((*R*)**-**/(*S*)**-C[4]B**) [48] and an achiral bis-thiazole orange (**bis-TO**) guest (Scheme 1). The exceptionally strong host–guest interactions between **C[4]B** and thiazole orange motifs enable stable supramolecular polymerization in aqueous solution at relatively low concentration, while simultaneously promoting ordered assembly and efficient chirality transfer. As a result, the achiral fluorophore is endowed with pronounced chiroptical activity, giving rise to strong circular dichroism and circularly polarized luminescence responses. The resulting supramolecular polymer exhibits red emission centered at approximately 625 nm together with significant CPL activity, with a luminescence dissymmetry factor (|*g*_lum_|) of 8.5 × 10^−3^, among the relatively high values reported for water-soluble host–guest CPL systems (Table S1). To the best of our knowledge, this is the first reported water-soluble supramolecular polymer based on host–guest interactions that exhibits CPL in the red-emission region. This work provides a general and facile strategy for constructing CPL-active supramolecular polymers in water through strong host–guest interactions, and highlights the potential of chiral macrocyclic hosts in chiroptical material design.

## 2. Materials and Methods

### 2.1. Materials

All reagents were purchased from commercial suppliers and used without further purification. N_3_-PEG-N_3_ (*M*_w_ = 600 Da, *Đ* = 1.02) was purchased from Jingpi Technology (Changchun, China). 4-Methylquinoline, 2-methylmercaptobenzothiazole, and copper(I) bromide (CuBr) were obtained from Sinopharm Chemical Reagent Co., Ltd (Shanghai, China). Alkylating agents (1-bromo-3-butyne and methyl iodide), sodium ascorbate (NaAsc), and *N*,*N*,*N*′,*N*′,*N*″-pentamethyldiethylenetriamine (PMDETA) were purchased from Macklin Biochemical Co., Ltd (Shanghai, China). All other solvents, including acetonitrile (ACN), ethanol (EtOH), and triethylamine (Et_3_N) were supplied by Aladdin Industrial Corporation (Shanghai, China).

### 2.2. Instruments and Equipment

Nuclear magnetic resonance (NMR) spectra were recorded on Bruker Avance III 400 spectrometers (Bruker, Karlsruhe, Germany), with working frequencies of 400 MHz for ^1^H, ^13^C and 2D diffusion-ordered NMR spectroscopy (DOSY). Mass spectra were recorded using Agilent 6540 TOF (HR-ESI-MS) (Agilent Technologies, Santa Clara, CA, USA). Fluorescence spectra were recorded in a conventional quartz cell (light path 10 mm) on spectrofluorometer Edinburgh FS5 (Edinburgh Instruments, Livingston, UK). UV-Vis spectra were recorded in a quartz cell (light path 10 mm) on a Cary 100 UV-Vis spectrophotometer equipped with a Cary dual cell peltier accessory (Agilent Technologies, Santa Clara, CA, USA). Dynamic Light Scattering (DLS) was measured using a Nano Brook Laser Scattering Spectrometer (Brookhaven Instruments, Holtsville, NY, USA). Scanning electron microscope (SEM) imaging was performed using an Apreo S LoVac from FEI Czech Co., Ltd (FEI Czech Co., Ltd, Brno, Czech Republic). Circular dichroism (CD) spectra were measured on MOS-450 of BioLogic (BioLogic, Claix, France). Circularly polarized luminescence (CPL) spectra were recorded on a JASCO CPL-300 (JASCO Corporation, Tokyo, Japan). Isothermal titration calorimetry (ITC) titration experiments were carried out on a PEAQ-ITC (Malvern Panalytical, Malvern, UK) at 298 K.

### 2.3. Synthetic Protocols

**The synthesis of (*R*)-/(*S*)-C[4]B:** (*R*)**-C[4]B** and (*S*)**-C[4]B** were prepared with reference to the previously reported method [48].

**The synthesis of A:** 4-Methylquinoline (0.51 g, 3.5 mmol) and 4-bromo-1-butyne (0.50 mL, 4.4 mmol) were added to a sealed tube and dissolved in ACN. The reaction mixture was heated at 80 °C for 8 h under sealed conditions. After cooling to room temperature naturally, the precipitate was collected by vacuum filtration and washed thoroughly to afford **A** (0.89 mg, 3.2 mmol, 90% yield) as a light gray solid. ^1^H NMR (400 MHz, DMSO-*d*_6_, 298 K) δ 9.43 (d, *J* = 6.1 Hz, 1H), 8.63 (d, *J* = 8.9 Hz, 1H), 8.56 (d, *J* = 8.5 Hz, 1H), 8.31–8.22 (m, 1H), 8.13 (d, *J* = 6.1 Hz, 1H), 8.10–8.03 (m, 1H), 5.20 (t, *J* = 6.6 Hz, 2H), 3.05 (t, *J* = 2.6 Hz, 1H), 3.03 (s, 3H), 2.99 (td, *J* = 6.6 Hz, 2H). ^13^C NMR (100 MHz, DMSO-*d*_6_, 298 K) δ: 159.4, 149.0, 136.6, 135.3, 129.7, 128.8, 127.3, 122.3, 119.3, 79.3, 75.6, 54.7, 19.8, 19.2. HR-ESI MS: calculated for C_14_H_14_N ([M−Br]^+^): 196.11207; found: 196.11231.

**The synthesis of TO-Alkyne:** Compound **B** was synthesized according to the protocols reported in the literature [49]. **A** (0.40 g, 1.4 mmol) and **B** (0.47 g, 1.4 mmol) were added to a 100 mL round-bottom flask and suspended in anhydrous ethanol. Triethylamine (Et_3_N) (2.0 mL, 14 mmol) was added dropwise under magnetic stirring, and the mixture gradually became a homogeneous red solution. The resulting reaction mixture was stirred continuously at 25 °C overnight to complete the reaction. The solvent was removed under reduced pressure, and the residue was washed with deionized water and filtered under vacuum to afford **TO-Alkyne** (0.56 g, 1.3 mmol, 95% yield) as a red solid. ^1^H NMR (400 MHz, DMSO-*d*_6_, 298 K) δ: 8.79 (d, *J* = 8.5 Hz, 1H), 8.61 (d, *J* = 7.3 Hz, 1H), 8.13 (d, *J* = 8.8 Hz, 1H), 8.05 (d, *J* = 7.9 Hz, 1H), 7.97 (t, *J* = 7.8 Hz, 1H), 7.80 (d, *J* = 8.4 Hz, 1H), 7.75 (t, *J* = 7.7 Hz, 1H), 7.61 (t, *J* = 7.9 Hz, 1H), 7.42 (t, *J* = 7.6 Hz, 1H), 7.34 (d, *J* = 7.2 Hz, 1H), 6.93 (s, 1H), 4.75 (t, *J* = 6.0 Hz, 2H), 4.03 (s, 3H), 3.02 (t, *J* = 2.0 Hz, 1H), 2.86 (m, 2H). ^13^C NMR (100 MHz, DMSO-*d*_6_, 298 K) δ: 160.4, 148.6, 144.8, 140.5, 136.8, 133.3, 128.2, 126.8, 125.9, 124.6, 124.1, 122.9, 117.9, 113.1, 107.3, 88.4, 80.0, 74.8, 52.0, 33.9, 18.6. HR-ESI MS: calculated for C_22_H_19_N_2_S ([M−Br]^+^): 343.12634; found: 343.12623.

**The synthesis of bis-TO**: N_3_-PEG-N_3_ (38 mg, 0.059 mmol), **TO-Alkyne** (50 mg, 0.118 mmol), CuBr (0.85 mg, 0.0059 mmol), NaAsc (2.3 mg, 0.012 mmol), and PMDETA (2.0 mg, 0.012 mmol) were added to a sealed tube and dissolved in DMF. The mixture was stirred at 50 °C for 5 h under a nitrogen atmosphere and protected from light. The solvent was removed under reduced pressure to yield **bis-TO** (75 mg, 0.050 mmol, 85% yield) as a reddish-brown viscous oil. ^1^H NMR (400 MHz, DMSO-*d*_6_, 298 K) δ: 8.79 (d, *J* = 8.3 Hz, 1H), 8.61 (d, *J* = 7.9 Hz, 1H), 8.13 (d, *J* = 8.7 Hz, 1H), 8.05 (d, *J* = 16.9 Hz, 2H), 7.97 (s, *J* = 7.5 Hz, 1H), 7.77 (m, 2H), 7.61 (m, 1H), 7.42 (t, *J* = 7.6 Hz, 1H), 7.34 (d, *J* = 8.0 Hz, 1H), 6.93 (s, 1H), 4.90 (t, 2H), 4.03 (s, 2H), 3.02 (t, *J* = 7.2 Hz, 3H), 2.86 (t, *J* = 7.0 Hz, 54H). ^13^C NMR (100 MHz, DMSO-*d*_6_, 298 K) δ: 160.4, 148.6, 144.8, 140.3, 133.9, 128.2, 127.6, 125.2, 124.9, 122.6, 117.9, 113.4, 107.3, 80.7, 69.7, 68.7, 58.3, 52.0, 29.0, 28.1, 26.6, 24.3. HR-ESI MS: calculated for C_70_H_90_N_10_O_12_S_2_ ([M−2Br]^2+^): 663.30851; found: 663.30719.

**Scheme 2:**
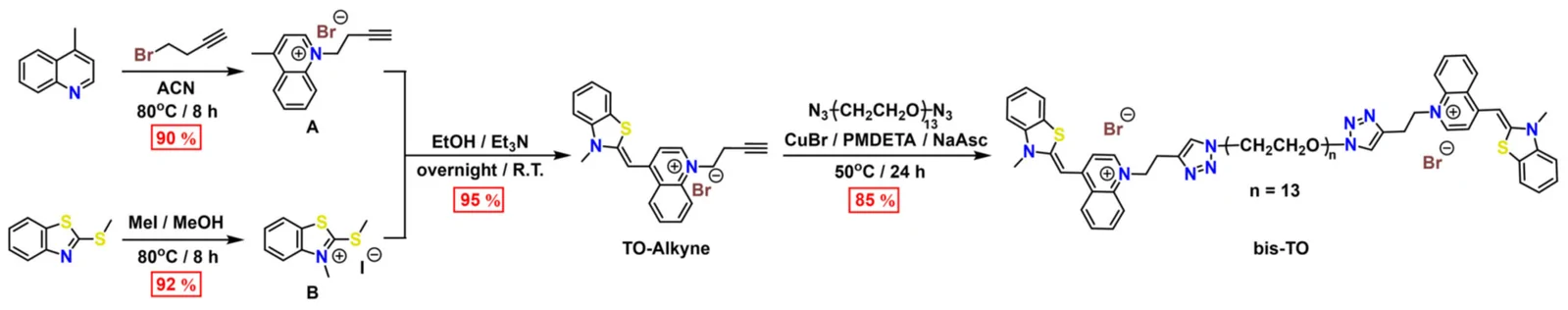
The synthesis route of **TO-Alkyne** and **bis-TO** (Red numbers denote yield values, R.T. denotes room temperature.).

The synthetic routes is illustrated in Scheme 2. The ^1^H NMR, ^13^C NMR and HR-ESI MS data are provided in the Supporting Information.

### 2.4. Preparation and Characterizations

#### 2.4.1. Fluorometric Titration

Fluorescence titrations were carried out on a fluorescence spectrophotometer at 298 K. Fluorometric titration experiments were performed by tracking the changes in fluorescence intensity of guest after titrating varied equivalents of (*S*)**-C[4]B**. The concentration of **TO-Alkyne** and **bis-TO** was fixed at 10.0 μM, while the concentration of (*S*)**-C[4]B** was gradually increased from 0 to 1.5 equiv. Fluorescence spectrometry conditions: excitation wavelength was set to 430 nm, slit width of 2.0/2.5 nm, the scanning range was set from 520 nm to 700 nm, and the emission wavelength was monitored at 620 nm. Each solution was equilibrated for 5 min before measurement, and all tests were performed in triplicate. And the *K*_a_ between the (*S*)**-C[4]B** and **TO-Alkyne** was determined according to the 1:2 binding stoichiometry. And the *K*_a_ between the (*S*)**-C[4]B** and **bis-TO** was determined according to the 1:1 binding stoichiometry.

Job’s plot obtained by recording the variation in fluorescence intensity at 626 nm for different ratios of (*S*)**-C[4]B** and **bis-TO** at a fixed total concentration of 10.0 μM.

#### 2.4.2. UV-Vis Titration

UV-Vis titrations were carried out at 298 K. The concentration of **TO-Alkyne** was fixed at 10.0 μM, while the concentration of (*S*)**-C[4]B** was gradually increased from 0 to 1.5 equiv. The scanning range was set from 400 nm to 600 nm, and absorption intensity was monitored at 525 nm. The concentration of **bis-TO** was fixed at 10.0 μM, while the concentration of (*S*)**-C[4]B** was gradually increased from 0 to 1.8 equiv. The scanning range was set from 400 nm to 600 nm, and absorption intensity was monitored at 475 nm. Each solution was equilibrated for 5 min before measurement, and all tests were performed in triplicate. And the Ka between the (*S*)**-C[4]B** and **TO-Alkyne** was determined according to the 1:2 binding stoichiometry. And the Ka between the (*S*)**-C[4]B** and **bis-TO** was determined according to the 1:1 binding stoichiometry.

#### 2.4.3. ITC Titration

Isothermal titration calorimetry (ITC) measurements were performed at 298 K. All solutions were prepared in H_2_O. The sample cell contained (*S*)**-C[4]B** solution (10.0 μM), and the syringe was loaded with **TO-Alkyne** solution (200 μM). 2.0 μL guest solution (the first injection was 0.40 μL) with 120 s spacing and 10 μcal/second reference power were injected 19 times into the ITC cell. The binding data was fitted using sequential binding sites in MicroCal PEAQ-ITC analysis software (version 1.52, Malvern Panalytical, Worcestershire, UK).

#### 2.4.4. SEM Measurements

SEM samples were prepared by dropping dilute aqueous solutions of **bis-TO**@(*S*)**-C[4]B** (10.0 μM and 1.0 mM) and **2TO-Alkyne**@(*S*)**-C[4]B** (10.0 μM) onto clean silicon wafers, respectively. The samples were air-dried naturally at room temperature and sputter-coated with platinum before observation.

#### 2.4.5. DOSY NMR

DOSY experiments were performed on a 400 MHz NMR spectrometer at 298 K. Samples were dissolved in D_2_O with a concentration of 0.5–5.0 mM. The diffusion time (Δ) and gradient length (δ) were optimized to achieve 80–90% signal attenuation.

#### 2.4.6. CD Spectra

CD spectra were recorded at 298 K in a 1 cm quartz cuvette. The scanning range was set from 200 nm to 600 nm. A baseline spectrum was first collected using pure water as a blank. Subsequently, spectra of (*R*)**-**/(*S*)**-C[4]B** and guest@(*R*)**-**/(*S*)**-C[4]B** complex were measured. The concentration of (*R*)**-**/(*S*)**-C[4]B** was fixed at 10.0 μM. **2TO-Alkyne**@(*R*)**-**/(*S*)**-C[4]B** homoternary complexes were prepared by mixing 10.0 μM host with 20.0 μM guest. **Bis-TO**@(*R*)**-**/(*S*)**-C[4]B** homoternary complexes were prepared by mixing 10.0 μM host with 10.0 μM guest.

#### 2.4.7. CPL Spectra

CPL spectra were recorded at 298 K in a 1 cm quartz cuvette. The scanning range was set from 520 nm to 700 nm. CPL spectra were recorded in H_2_O for the host–guest complexes formed between (*R*)**-**/(*S*)**-C[4]B** and **TO-Alkyne** or **bis-TO**. **2TO-Alkyne**@(*R*)**-**/(*S*)**-C[4]B** homoternary complexes were prepared by mixing 10.0 μM host with 20.0 μM guest. **Bis-TO**@(*R*)**-**/(*S*)**-C[4]B** homoternary complexes were prepared by mixing 10.0 μM host with 10.0 μM guest.

## 3. Results and Discussion

### 3.1. 1:2 Binding Between (S)-C*[4]*B and TO-Alkyne in Water

In our previous study, thiazole orange (**TO**) was demonstrated to exhibit ultrastrong 1:2 host–guest complexation with (*S*)**-C[4]B**, with an overall binding constant (*K*_12_) of 8.4 × 10^16^ M^−2^ as determined by ITC. To introduce a reactive site for subsequent polymerization, an alkyne-functionalized derivative of **TO** (**TO-Alkyne**) was synthesized, and its host–guest interactions with (*S*)**-C[4]B** were systematically investigated.

UV-Vis absorption and fluorescence titrations were first conducted to examine the complexation behavior between (*S*)**-C[4]B** and **TO-Alkyne**. Since (*S*)**-C[4]B** itself exhibits noticeable UV absorption and fluorescence emission, its potential interference was evaluated prior to the titration experiments. As shown in Figure S9, although partial overlap exists between the absorption and emission spectra of (*S*)**-C[4]B** and **TO-Alkyne**, sufficient spectral windows remain available for monitoring the formation of host–guest complexes.

**Figure 1:**
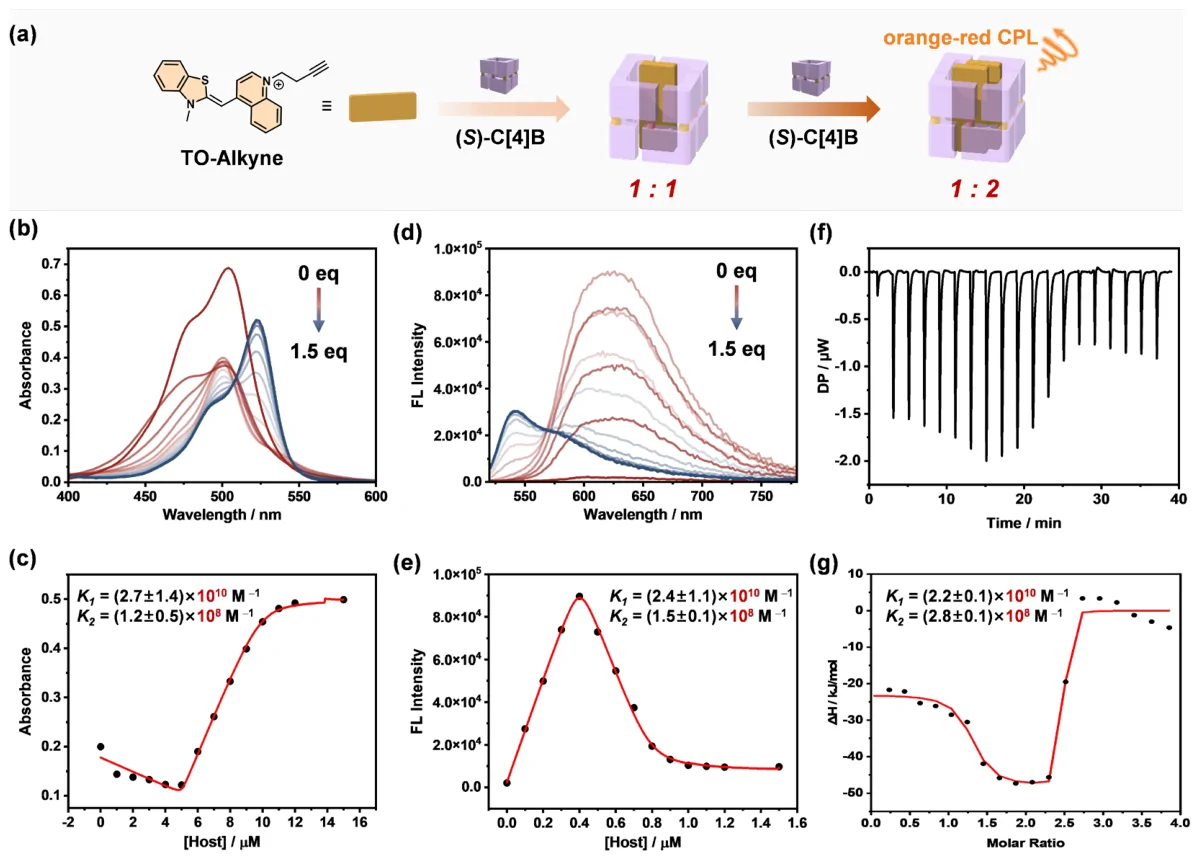
(**a**) Schematic illustration of the formation process of **2TO-Alkyne**@(*S*)**-C[4]B** homoternary complex. (**b**) UV-Vis titration spectra obtained by adding (*S*)**-C[4]B** (up to 15.0  μM) into a 10.0  μM solution of **TO-Alkyne** at 298 K. (**c**) The fitting curve plot by tracking absorbance at *λ* = 525 nm. (**d**) Fluorometric titration spectra obtained by adding (*S*)**-C[4]B** (up to 15.0 μM) into a 10.0 μM solution of **TO-Alkyne** at 298 K. (**e**) The fitting curve plot by tracking emission intensity at *λ*  =  620 nm, with the nonlinear curve fitting by using a 1:2 host–guest binding model. (**f**) Isothermal titration calorimetry (ITC) isotherms obtained by adding **TO-Alkyne** (200  μM) into a 10.0  μM solution of (*S*)**-C[4]B** at 298 K. (**g**) The analyzed data of ITC isotherms with the sequential binding model.

As shown in Figure 1b, upon gradual addition of (*S*)**-C[4]B** to an aqueous solution of **TO-Alkyne** (10.0 μM), the absorption band centered at 504 nm progressively decreased, accompanied by the emergence and growth of a new band at 525 nm. The pronounced bathochromic shift indicates a substantial change in the electronic environment of **TO-Alkyne** upon complexation with (*S*)**-C[4]B**. Notably, analysis of the absorbance variation at 525 nm revealed two distinct inflection points (Figure 1c), suggesting the existence of two sequential binding modes during the titration process, corresponding to the formation of 1:2 and 1:1 host–guest complexes. Fitting the titration data using a 1:2 binding model afforded an overall binding constant of *K*_12_ = 3.2 × 10^18^ M^−2^, with *K*_1_ = (2.7 ± 1.4) × 10^10^ M^−1^ and *K*_2_ = (1.2 ± 0.5) × 10^8^ M^−1^.

The fluorescence titration experiments further supported the formation of both 1:2 and 1:1 host–guest complexes (Figure 1d,e). During the titration, the emission band at approximately 625 nm initially increased dramatically and subsequently decreased, while a new emission band centered at around 540 nm gradually appeared and became dominant. At low host concentrations, the formation of the 1:2 complex 2**TO-Alkyne**@(*S*)**-C[4]B** resulted in a significant enhancement of the 625 nm emission, which can be attributed to the restriction of intramolecular rotations and vibrations of **TO-Alkyne** upon complexation, thereby suppressing non-radiative decay pathways. Upon further addition of (*S*)**-C[4]B**, the dominant binding species gradually evolved from the 1:2 complex to the 1:1 complex as the host concentration increased, accompanied by the appearance of a blue-shifted emission band around 540 nm. By monitoring the fluorescence intensity changes at 620 nm and fitting the data with a 1:2 binding model, an overall binding constant of *K*_12_ = 3.6 × 10^18^ M^−2^ was obtained, with *K*_1_ = (2.4 ± 1.1) × 10^10^ M^−1^ and *K*_2_ = (1.5 ± 0.1) × 10^8^ M^−1^. Further evidence for the stepwise complexation process was provided by ITC measurements. As shown in Figure 1f,g, the ITC titration curve revealed two distinct transition regions, indicative of the sequential formation of host–guest complexes. Nonlinear fitting of the ITC data using a sequential binding model yielded an overall binding constant of *K*_12_ = 6.2 × 10^18^ M^−2^, with *K*_1_ = (2.2 ± 0.1) × 10^10^ M^−1^ and *K*_2_ = (2.8 ± 0.1) × 10^8^ M^−1^.

Collectively, the results obtained from UV-Vis spectroscopy, fluorescence titration, and ITC consistently demonstrate that (*S*)**-C[4]B** and **TO-Alkyne** undergo stepwise host–guest complexation through both 1:2 and 1:1 binding modes. The exceptionally strong binding affinity between the host and guest provides a robust foundation for the subsequent construction of linear supramolecular polymers in aqueous solution.

### 3.2. Formation and Characterization of the Supramolecular Polymer in Water

Through a copper-catalyzed azide-alkyne cycloaddition (CuAAC) reaction, **TO-Alkyne** was covalently linked to a polyethylene glycol (PEG, *M*_w_ = 600 Da, *D* = 1.02) chain to afford a PEG-based guest functionalized with thiazole orange (**TO**) moieties at both termini (**bis-TO**). To avoid possible intramolecular 1:1 complexation, a long and flexible oligo (ethylene glycol) linker (13 repeating units) was incorporated between the two TO motifs. The reduced effective molarity of the tethered guest disfavors intramolecular binding and promotes intermolecular host–guest interactions, thereby facilitating the formation of linear supramolecular polymers. Owing to the ultrastrong 1:2 host–guest complexation between (*S*)**-C[4]B** and **TO-Alkyne**, we envisioned that a single chiral (*S*)**-C[4]B** host could simultaneously bind two **TO** units from adjacent **bis-TO** molecules in a head-to-tail manner, thereby promoting the formation of linear supramolecular polymers in aqueous solution.

**Figure 2:**
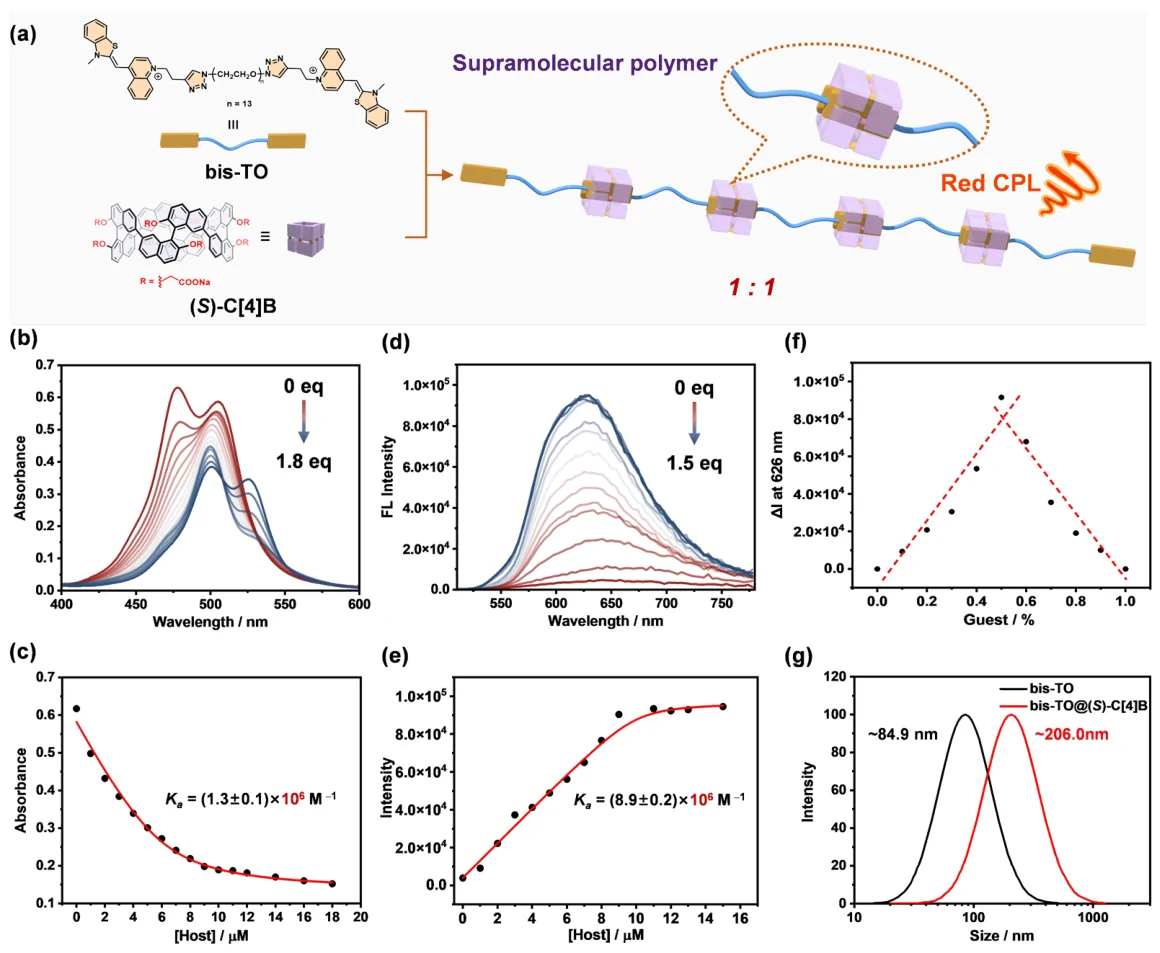
(**a**) Schematic illustration of the formation of the **bis-TO**@(*S*)**-C[4]B** supramolecular polymer. (**b**) UV-Vis titration spectra obtained by adding (*S*)**-C[4]B** (up to 18.0  μM) into a 10.0  μM solution of **bis-TO** at 298 K. (**c**) The fitting curve plot by tracking absorbance at *λ* = 475 nm. (**d**) Fluorometric titration spectra obtained by adding (*S*)**-C[4]B** (up to 15.0 μM) into a 10.0 μM solution of **bis-TO** at 298 K. (**e**) The fitting curve plot by tracking emission intensity at *λ * =  620 nm, with the nonlinear curve fitting by using a 1:1 host–guest binding model. (**f**) The Job’s plot obtained by tracking the variation in the fluorescence emission at 626 nm for different ratios of (*S*)**-C[4]B** and **bis-TO** at a fixed total concentration of 10.0 μM. (**g**) The size of the guest (black line, PDI = 0.166) and supramolecular polymer (red line, PDI = 0.228) at 5.0 μM were characterized by DLS.

The host–guest complexation behavior between (*S*)**-C[4]B** and **bis-TO** was first investigated by UV-Vis absorption and fluorescence titrations. As shown in Figure 2b, upon gradual addition of (*S*)**-C[4]B**, the two characteristic absorption bands of **bis-TO** at 478 nm and 505 nm progressively decreased, accompanied by the emergence of a new absorption band centered at 525 nm. The pronounced bathochromic shift indicates a substantial change in the electronic environment of the TO chromophores upon host–guest complexation. This spectral evolution can be attributed to the encapsulation of the **TO** units within the cavity of (*S*)**-C[4]B**, which restricts their intramolecular rotational motions and stabilizes the conjugated electronic structure. Consistent with the UV-Vis results, fluorescence titration revealed a dramatic enhancement of the emission band centered at approximately 625 nm upon addition of (*S*)**-C[4]B** (Figure 2d).

By monitoring the absorbance changes at 475 nm and the fluorescence intensity changes at 620 nm, the binding isotherms could be satisfactorily fitted using a 1:1 binding model, indicating that (*S*)**-C[4]B** and **bis-TO** predominantly form a 1:1 host–guest complex. The corresponding *K*_a_ were determined to be (1.3 ± 0.1) × 10^6^ M^−1^ and (8.9 ± 0.2) × 10^6^ M^−1^ from UV-Vis and fluorescence titrations, respectively (Figure 2c,e). Furthermore, Job’s plot analysis provided additional evidence for the 1:1 binding stoichiometry (Figure 2f).

The formation of larger supramolecular assemblies was further confirmed by dynamic light scattering (DLS). As shown in Figure 2g, the average hydrodynamic diameter of the **bis-TO**@(*S*)**-C[4]B** assembly in aqueous solution was approximately 2.4 times larger than that of free **bis-TO**, indicating the formation of higher-order supramolecular aggregates. It is noteworthy that free **bis-TO** also exhibited a relatively large hydrodynamic diameter, which is likely attributable to the self-aggregation in aqueous solution. The PDI values of the free **bis-TO** and the polymer assembly are 0.166 and 0.228, respectively, both below 0.3, confirming relatively uniform size distributions; the slight increase in PDI is consistent with the inherent polydispersity of linear polymer chains.

**Figure 3:**
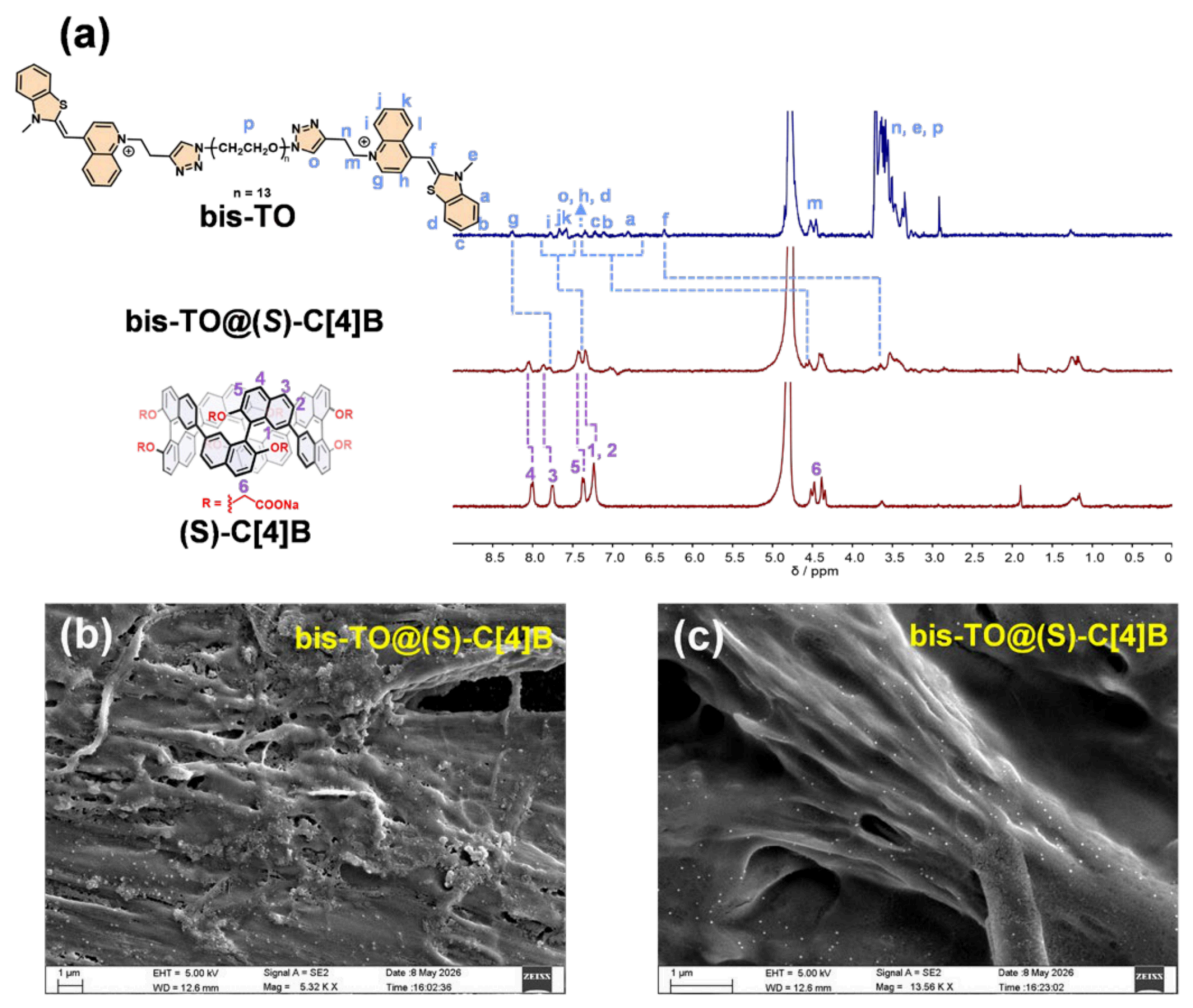
(**a**) ^1^H NMR spectra of **bis-TO**, **bis-TO**@(*S*)**-C[4]B** and (*S*)**-C[4]B** in D_2_O at 298 K (400 MHz, [**bis-TO**] = [(*S*)**-C[4]B**] = 1.0 mM). SEM images of **bis-TO**@ (*S*)**-C[4]B** at concentrations of (**b**) 10.0 μM and (**c**) 1.0 mM.

The supramolecular polymer was characterized by ^1^H NMR spectroscopy in D_2_O. As shown in Figure 3a, upon addition of (*S*)**-C[4]B** to **bis-TO**, significant signal broadening accompanied by obvious chemical shift changes was observed, indicating the formation of host–guest polymeric species in solution. The proton signals corresponding to the thiazole orange moieties of **bis-TO** exhibited pronounced upfield shifts upon complexation with (*S*)**-C[4]B** relative to those of the free guest, suggesting a strong shielding effect exerted by the host cavity on the encapsulated guest protons. Meanwhile, the different positions occupied by individual protons within the cavity resulted in varying degrees of shielding, leading to distinct chemical shift changes.

The polymerization process was further investigated using two-dimensional diffusion-ordered spectroscopy (DOSY NMR). As the concentrations of the host and guest were increased from 0.5 mM to 5.0 mM, the measured weighted average diffusion coefficient (*D*) decreased significantly from 2.74 × 10^−10^ m^2^ s^−1^ to 1.53 × 10^−10^ m^2^ s^−1^ (Figures S10–S15). The nearly two-fold decrease in the diffusion coefficient with increasing concentration provides clear evidence for concentration-dependent supramolecular polymerization. It is worth noting that the high association constant (*K*_a_ ~ 10^6^ M^−1^) of the **C[4]B/bis-TO** host–guest interaction provides a strong driving force for supramolecular polymerization from a theoretical perspective, favoring the formation of long chains with high degrees of polymerization (DP) at appropriate concentrations and stoichiometric ratios. However, the actual DP is highly sensitive to the host/guest stoichiometric ratio, and even small deviations from the ideal ratio can significantly affect the average chain length. Moreover, the absolute molecular weight of dynamic supramolecular polymers is difficult to determine directly. Although DLS, DOSY NMR, and microscopic characterization can provide information on the size and morphology of the assemblies, they do not allow an accurate determination of the absolute DP.

Furthermore, the assembly morphology of the supramolecular polymer was examined by scanning electron microscopy (SEM). As shown in Figure 3b,c, the assemblies formed at different concentrations displayed slightly helical linear polymeric structures accompanied by further aggregation. However, the 2**TO-Alkyne**@(*S*)**-C[4]B** host–guest complex exhibited an amorphous morphology. These observations indicate that the host–guest interactions between **bis-TO** and (*S*)**-C[4]B** drive the formation of one-dimensional linear supramolecular polymers, which subsequently undergo higher-order aggregation.

### 3.3. The Chiroptical Properties of the Supramolecular Polymer in Water

The pronounced chiroptical properties of the host encouraged us to further investigate whether its chirality could be transferred to the guest and ultimately expressed in the resulting supramolecular polymer. We first examined the feasibility of chirality transfer in the 1:2 host–guest complex formed between chiral (*R*)**-**/(*S*)**-C[4]B** and **TO-Alkyne**. As shown in Figure S16, upon addition of (*R*)**-**/(*S*)**-C[4]B** to **TO-Alkyne**, the formation of 2**TO-Alkyne**@(*R*)**-**/(*S*)**-C[4]B** gave rise to distinct induced CD signals. In particular, new CD bands appeared in the 400–600 nm region, where the achiral **TO-Alkyne** guest absorbs, indicating efficient chirality transfer from the chiral host to the bound guest. Consistently, CPL emission centered at approximately 600 nm was also observed. These results clearly demonstrate that the chiral **C[4]B** host can effectively induce chiroptical activity in an otherwise achiral guest upon complexation.

**Figure 4:**
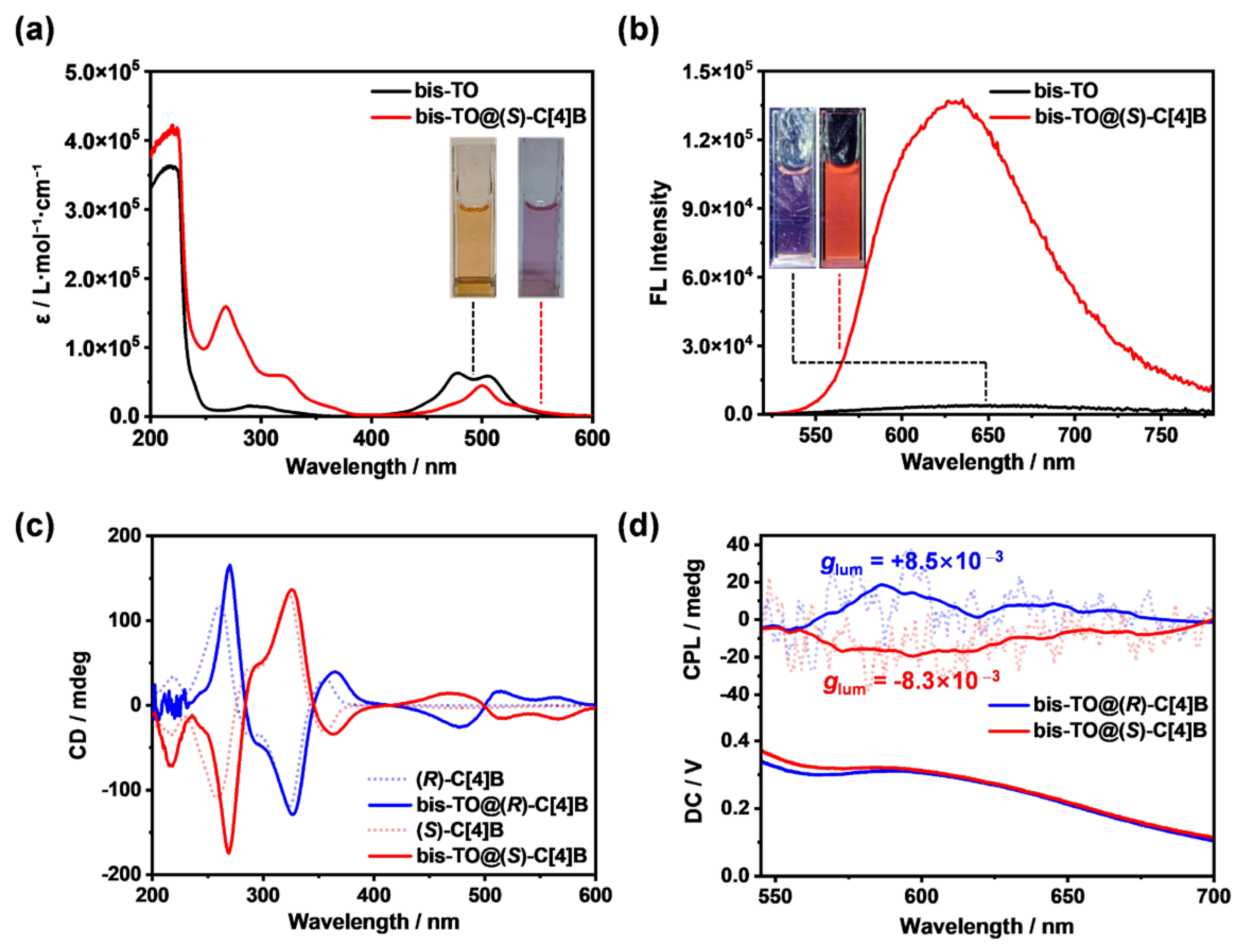
(**a**) UV-Vis spectra, (**b**) Fluorescence spectra (H_2_O, 10.0 μM, 298 K) of **bis-TO**, **bis-TO**@(*S*)**-C[4]B**. (**c**) CD spectra and (**d**) CPL spectra (H_2_O, 10.0 μM, 298 K) of (*R*)**-**/(*S*)**-C[4]B**, **bis-TO**@ (*R*)**-**/(*S*)**-C[4]B**.

On this basis, we further investigated the chiroptical properties of the supramolecular polymer assembled from **bis-TO** and (*R*)**-**/(*S*)**-C[4]B** in aqueous solution. As shown in Figure 4c, the chiral hosts (*R*)**-**/(*S*)**-C[4]B** displayed mirror-image Cotton effects in the 200–400 nm region. After equimolar assembly with the achiral **bis-TO** guest, the resulting **bis-TO**@(*R*)**-**/(*S*)**-C[4]B** supramolecular polymers exhibited enhanced CD signals in the 200–400 nm region, together with new mirror-image Cotton effects in the 400–600 nm region. The latter can be attributed to chirality transfer from the chiral host framework to the achiral **bis-TO** chromophores within the polymeric assembly. CPL measurements further confirmed the successful chiral induction in the supramolecular polymer. The **bis-TO**@(*R*)**-C[4]B** and **bis-TO**@(*S*)**-C[4]B** assemblies showed clear CPL emission at around 600 nm, in good agreement with their fluorescence spectra, with luminescence dissymmetry factors (|*g*_lum_|) of +8.5 × 10^−3^ and −8.3 × 10^−3^, respectively. The chirality transfer from chiral **C[4]B** to the achiral **TO** units is likely associated with the dissymmetric microenvironment and conformational restriction induced by host–guest complexation. The strong host–guest interactions confine the **TO** chromophore within the chiral cavity of **C[4]B**, exposing it to a chiral microenvironment and favoring a specific spatial conformation. Such asymmetric and conformationally restricted binding provides a plausible pathway for transferring the inherent chirality of **C[4]B** to the achiral **TO** chromophore, consistent with the observed induced CD and CPL signals. In the supramolecular polymer, the ordered arrangement of adjacent **TO** units may further contribute to the chiroptical response through excitonic interactions. Nevertheless, the individual contributions of these effects cannot be unambiguously distinguished based on the current experimental data.

These results demonstrate that the formation of the host–guest supramolecular polymer preserves the favorable CPL-emissive properties of the complex. We also summarized several representative examples of water-soluble supramolecular assemblies exhibiting CPL activity, as shown in Table S1. The comparison indicates that achieving red-emissive CPL materials solely through host–guest interactions in aqueous media remains relatively challenging, with most reported studies still focusing on the blue-to-yellow emission region. Therefore, it is worth noting that the obtained |*g*_lum_| values are among the relatively high levels reported for CPL-active host–guest supramolecular polymers [17].

## 4. Conclusions

In summary, we have constructed a water-soluble chiral luminescent supramolecular polymer through host–guest complexation between chiral macrocycles ((*R*)**-**/(*S*)**-C[4]B**) and an achiral bis-thiazole orange guest (**bis-TO**). **C[4]B** shows exceptionally strong binding toward the thiazole orange moiety, with an overall 1:2 binding constant of up to 10^18^ M^−2^, thereby enabling efficient supramolecular polymerization in aqueous solution. The formation of one-dimensional polymeric assemblies was supported by DLS, DOSY NMR, and SEM analyses. Benefiting from the chiral macrocyclic framework and strong host–guest recognition, chirality was efficiently transferred to the achiral **bis-TO** chromophores, giving rise to pronounced induced CD and CPL responses. The resulting supramolecular polymer exhibited red fluorescence and notable CPL activity, with a |*g*_lum_| value of approximately 8.5 × 10^−3^. This work demonstrates that ultrastrong host–guest recognition is an effective strategy for integrating aqueous supramolecular polymerization with chirality transfer, providing a useful platform for constructing CPL-active supramolecular materials in water.

## Data Availability

The data presented in this study are available on request from the corresponding author.

## Supplementary Materials

The following supporting information can be downloaded at https://www.mdpi.com/article/10.3390/polym18172040/s1: Figure S1. ^1^H NMR spectrum of **A** (DMSO-*d_6_*, 400 MHz, 298K). Figure S2. ^13^C NMR spectrum of **A** (DMSO-*d_6_*, 100 MHz, 298K). Figure S3. HR-ESI mass spectrum of **A**. Figure S4. ^1^H NMR spectrum of **TO-Alkyne** (DMSO-*d_6_*, 400 MHz, 298K). Figure S5. ^13^C NMR spectrum of **TO-Alkyne** (DMSO-*d_6_*, 100 MHz, 298K). Figure S6. HR-ESI mass spectrum of **TO-Alkyne**. Figure S7. ^1^H NMR spectrum of **bis-TO** (DMSO-*d_6_*, 400 MHz, 298K). Figure S8. ^13^C NMR spectrum of **C** (DMSO-*d_6_*, 100 MHz, 298K). Figure S9. HR-ESI mass spectrum of **bis-TO**. Figure S10. (a) UV-Vis absorption spectra of (*S*)**-C[4]B**, **TO-Alkyne** and **bis-TO** (H_2_O, 10.0 μM, 298 K). (b) Fluorescence Emission spectra of (*S*)**-C[4]B**, **TO-Alkyne** and **bis-TO** (H_2_O, 10.0 μM, 298 K). Figure S11. Concentration-dependent changes in diffusion coefficient values (*D*) of the **bis-TO**@(*S*)**-C[4]B** supramolecular polymer determined by DOSY NMR (D_2_O, 298 K). Figure S12. DOSY spectrum of **bis-TO**@(*S*)**-C[4]B** (298 K, 0.50 × 10^−3^ M in D_2_O). Figure S13. DOSY spectrum of **bis-TO**@(*S*)**-C[4]B** (298 K, 1.0 × 10^−3^ M in D_2_O). Figure S14. DOSY spectrum of **bis-TO**@(*S*)**-C[4]B** (298 K, 2.0 × 10^−3^ M in D_2_O). Figure S15. DOSY spectrum of **bis-TO**@(*S*)**-C[4]B** (298 K, 3.0 × 10^−3^ M in D_2_O). Figure S16. DOSY spectrum of **bis-TO**@(*S*)**-C[4]B** (298 K, 5.0 × 10^−3^ M in D_2_O). Figure S17. SEM images of **2TO-Alkyne**@(*S*)**-C[4]B** at concentration of 10.0 μM. Figure S18. (a) UV-Vis spectra, (b) Fluorescence spectra (H_2_O, 10.0 μM, 298 K) of **TO-Alkyne**, 2**TO-Alkyne**@(*S*)**-C[4]B**. (c) CD spectra and (d) CPL spectra (H_2_O, 10.0 μM, 298 K) of (*R*)**-**/(*S*)**-C[4]B**, 2**TO-Alkyne**@(*R*)**-**/(*S*)**-C[4]B**. Table S1. The *g*_lum_ values for CPL emission of aqueous chiral supramolecular assemblies.
