# Supplementary material for: Ultrastrong Host–Guest Recognition Driven Chiral Supramolecular Polymers with Circularly Polarized Luminescence in Water
Source: Polymers (Basel). 2026 Aug 22;18(17):2040. doi: 10.3390/polym18172040 (PMC13568129; doi:10.3390/polym18172040)
Supplement: Supplementary file 1 [file polymers-18-02040-s001.zip › polymers-4446979-supplementary.pdf]

Supporting Information

Ultrastrong Host-Guest Recognition Driven Chiral Supramolecular Polymers with Circularly Polarized Luminescence in Water

Ya-Ping Chen †, Si-Dan Guo †, Jinlei Zhou, Xiaoyu Luo and Kang Cai *

State Key Laboratory of Elemento-Organic Chemistry, College of Chemistry, Frontiers Science Center for New Organic Matter, Nankai University, Tianjin 300071, China; 2120241198@mail.nankai.edu.cn (Y.-P.C.); 1120220495@mail.nankai.edu.cn (S.-D.G.); zjl201226@163.com (J.Z.); 1120250686@mail.nankai.edu.cn (X.L.)

* Correspondence: kangcai@nankai.edu.cn

† These authors contributed equally to this work.

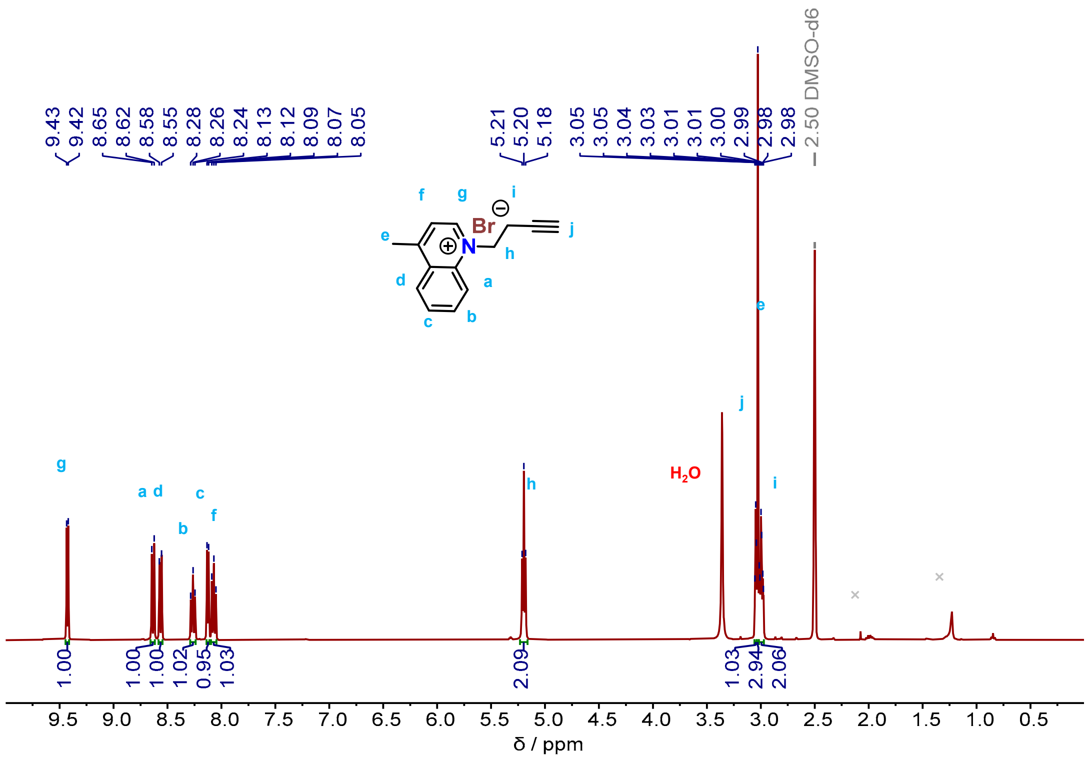

Figure S1. ^{1}H NMR spectrum of **A** (DMSO-*d*$_6$, 400 MHz, 298K).

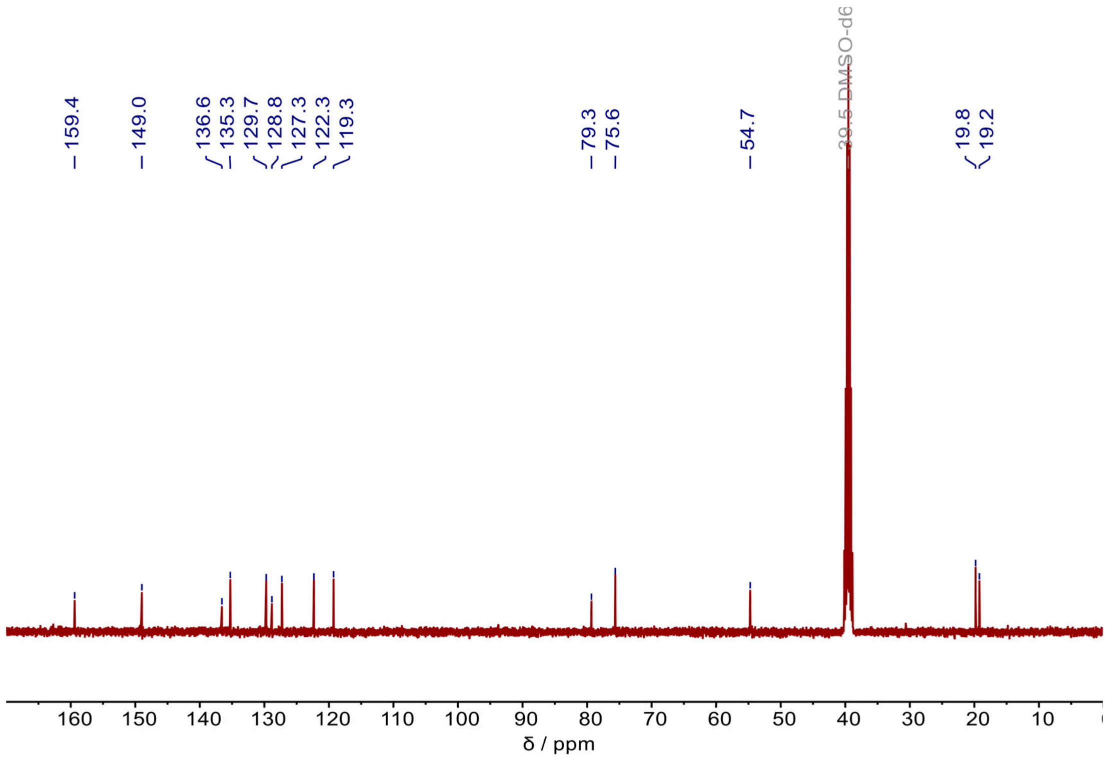

Figure S2. ^{13}C NMR spectrum of **A** (DMSO-*d*$_6$, 100 MHz, 298K).

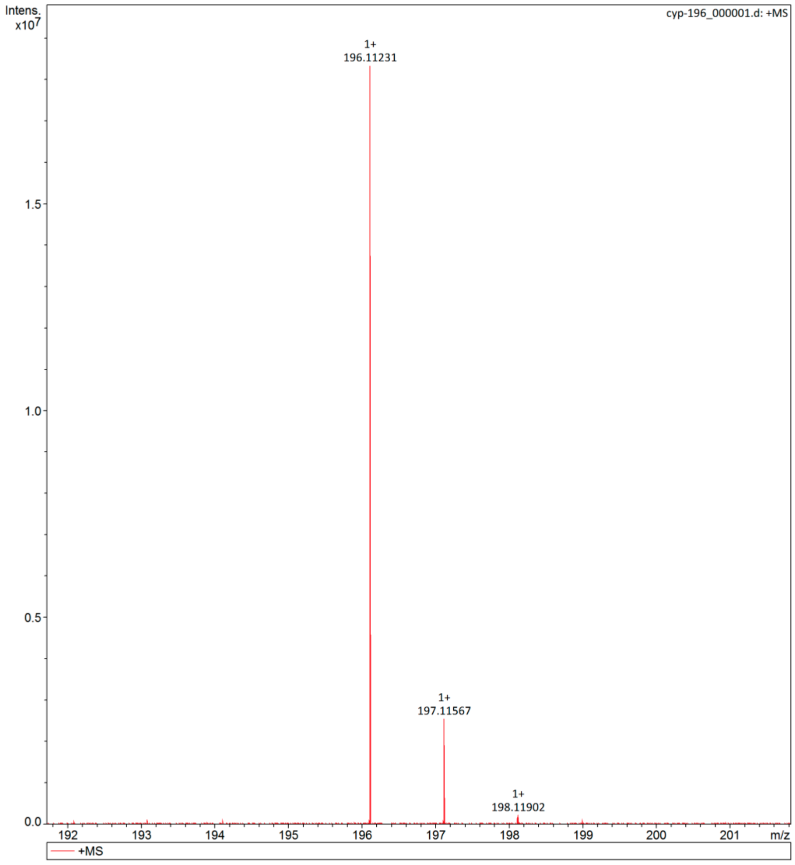

Figure S3. HR-ESI mass spectrum of **A**.

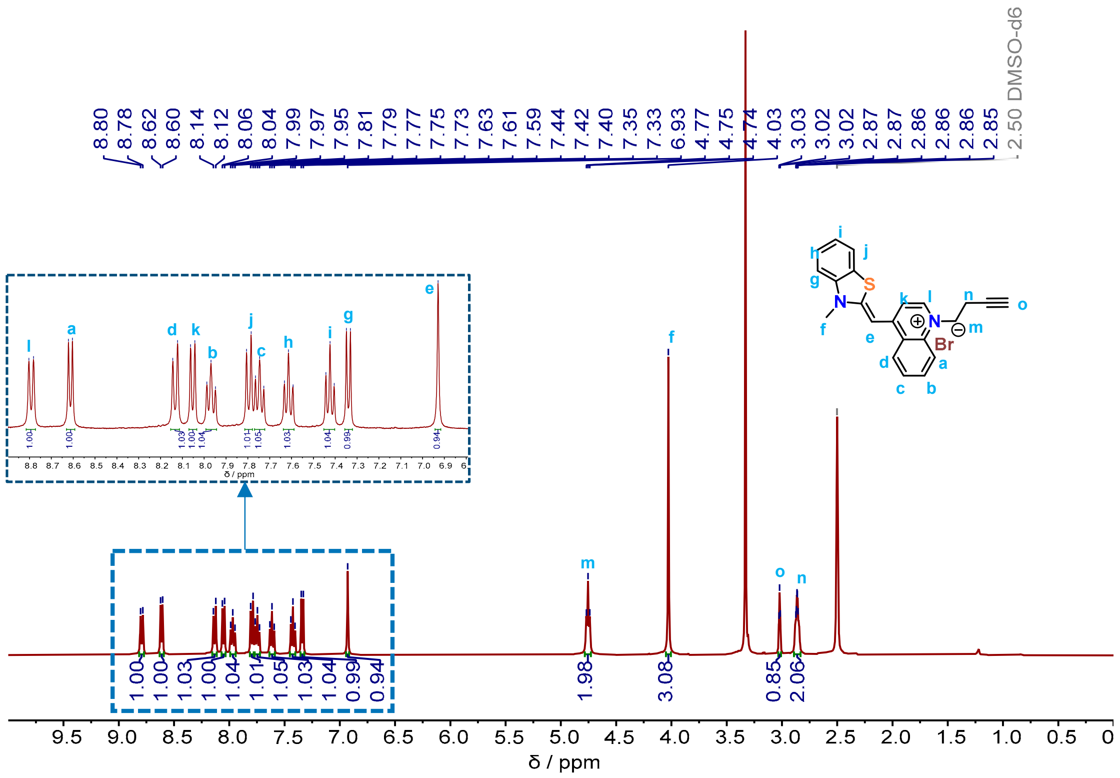

Figure S4. ^{1}H NMR spectrum of **TO-Alkyne** (DMSO-*d6*, 400 MHz, 298K).

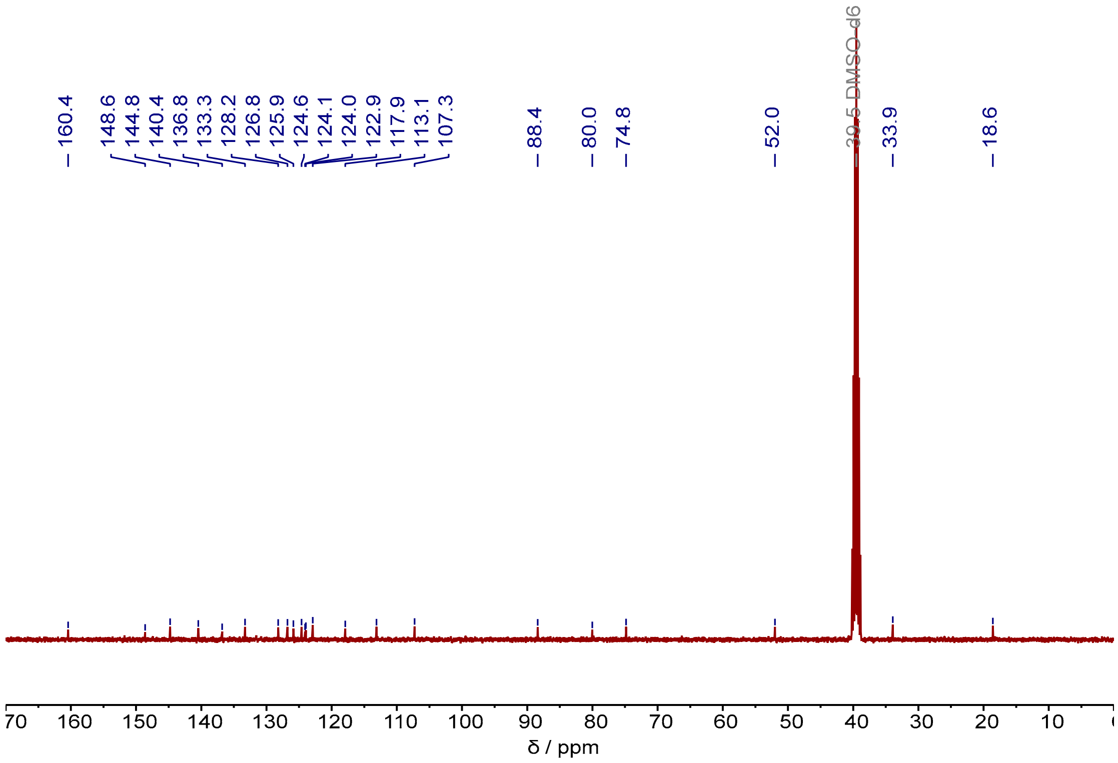

Figure S5. ^{13}C NMR spectrum of **TO-Alkyne** (DMSO-d_6, 100 MHz, 298K).

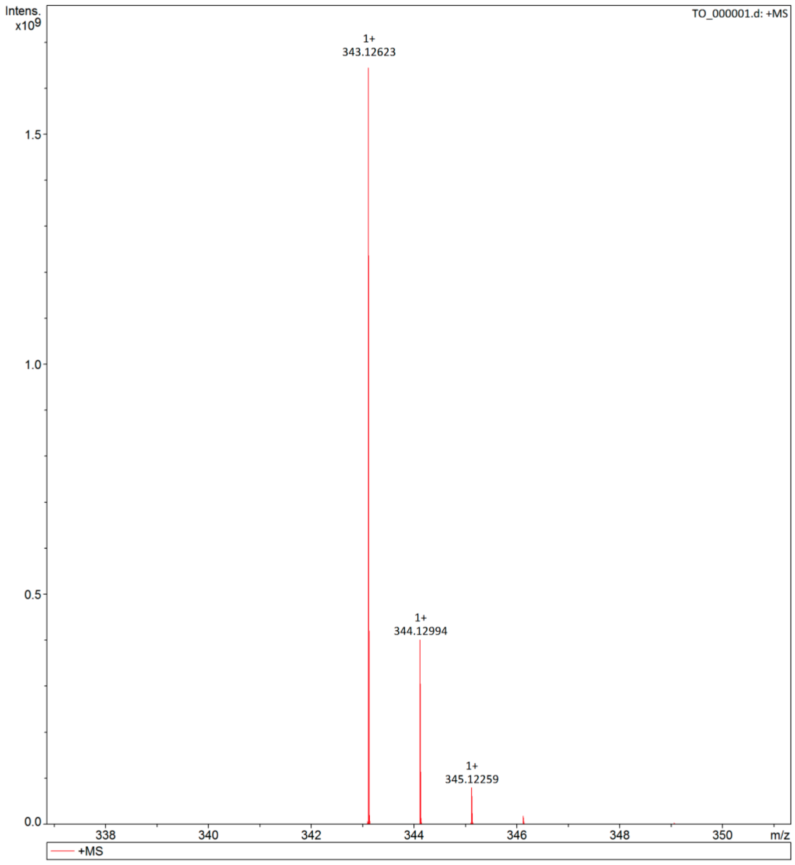

Figure S6. HR-ESI mass spectrum of **TO-Alkyne**.

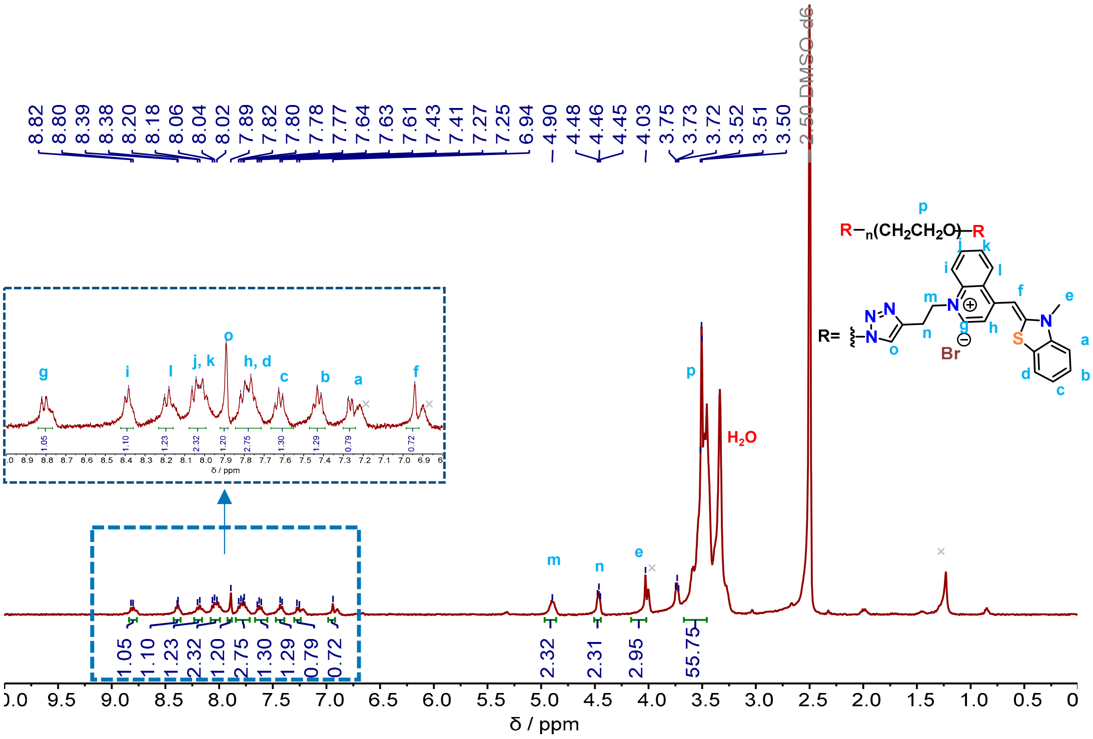

Figure S7. ^{1}H NMR spectrum of **bis-TO** (DMSO-*d*$_6$, 400 MHz, 298K).

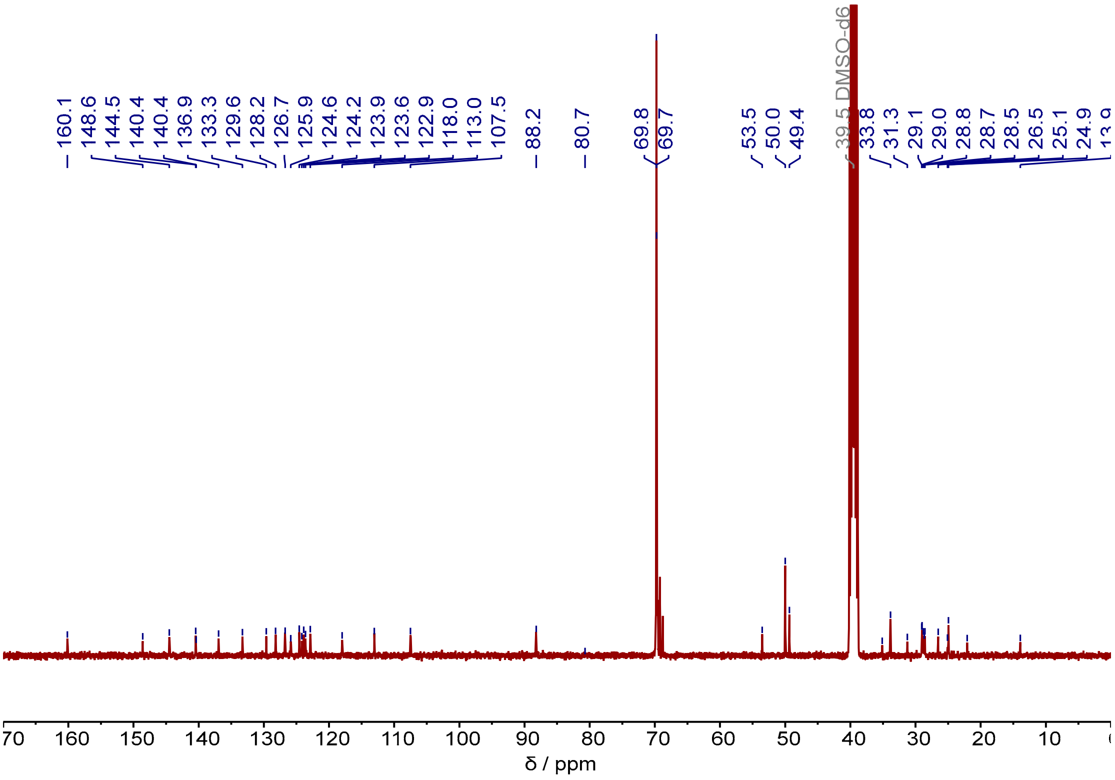

Figure S8. ^{13}C NMR spectrum of **bis-TO** (DMSO-*d*$_6$, 100 MHz, 298K).

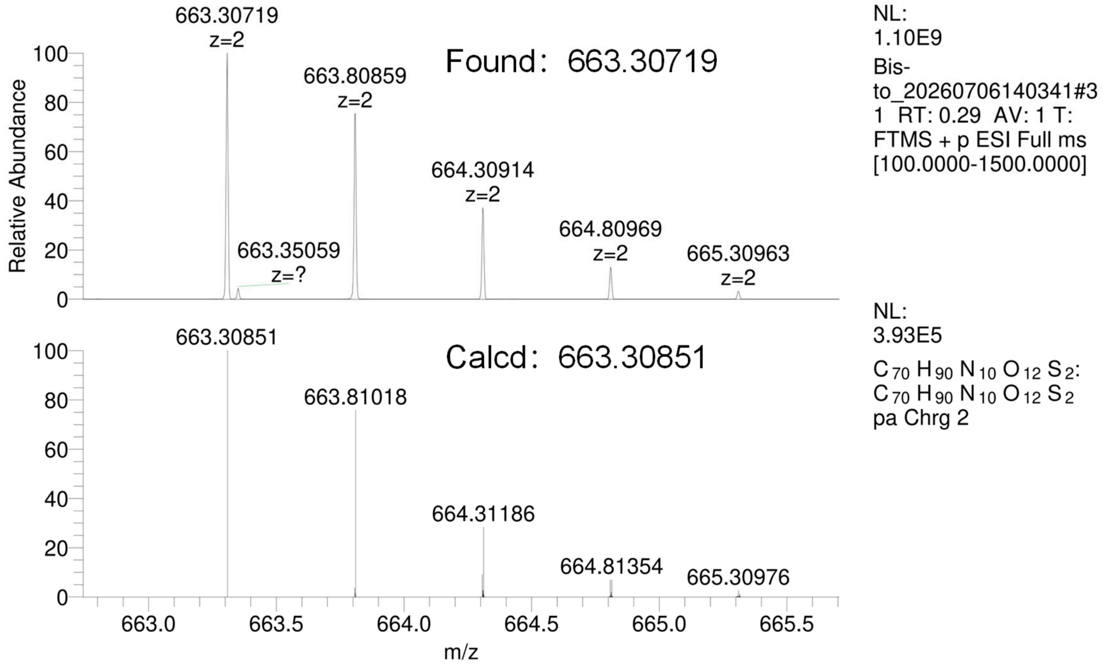

Figure S9. HR-ESI mass spectrum of **bis-TO**.

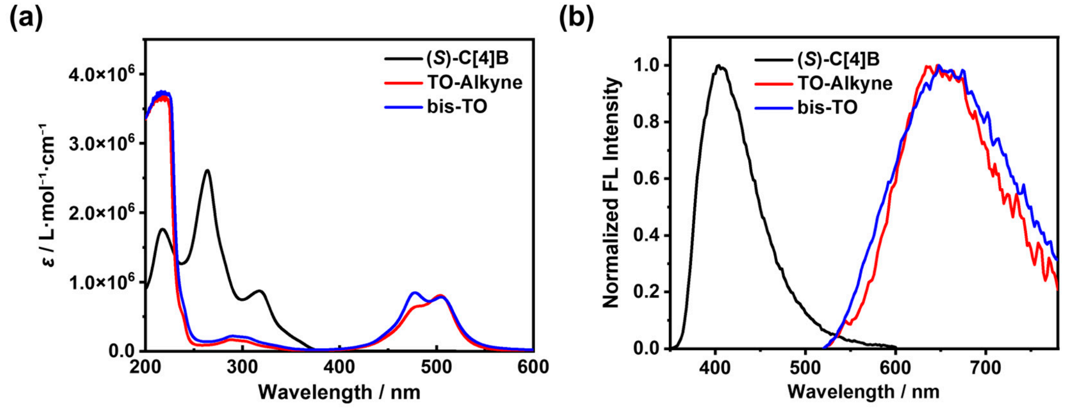

Figure S10. (a) UV-Vis absorption spectra of (*S*)**-C[4]B**, **TO-Alkyne** and **bis-TO** (H_2O, 10.0 μM, 298 K). (b) Fluorescence Emission spectra of (*S*)**-C[4]B**, **TO-Alkyne** and **bis-TO** (H_2O, 10.0 μM, 298 K).

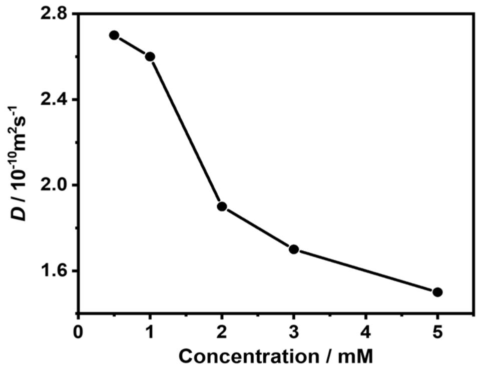

Figure S11. Concentration-dependent changes in diffusion coefficient values (*D*) of the **bis-TO@(*S*)-C[4]B** supramolecular polymer determined by DOSY NMR (D_2O, 298 K).

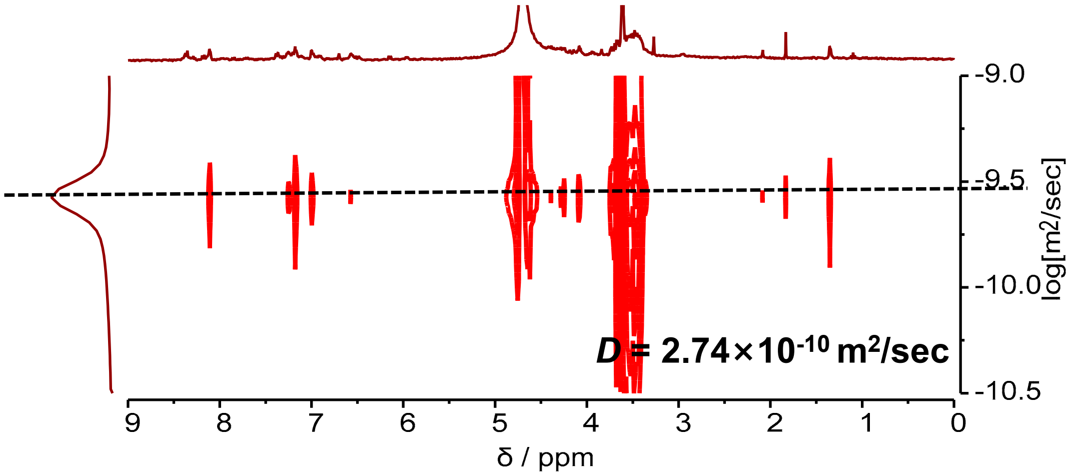

Figure S12. DOSY spectrum of **bis-TO@(*S*)-C[4]B** (298 K, 0.50×10^{-3} M in D_2O).

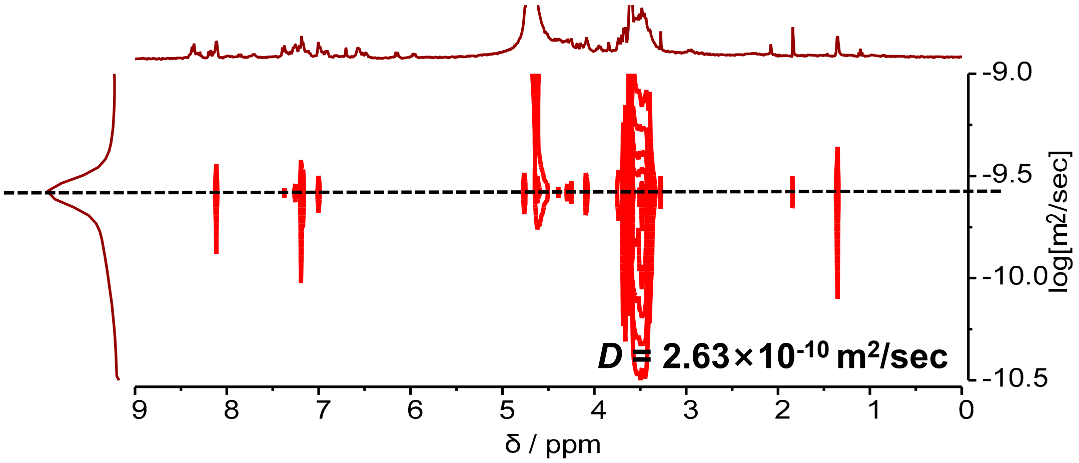

Figure S13. DOSY spectrum of **bis-TO@(*S*)-C[4]B** (298 K, 1.0×10^{-3} M in D_2O).

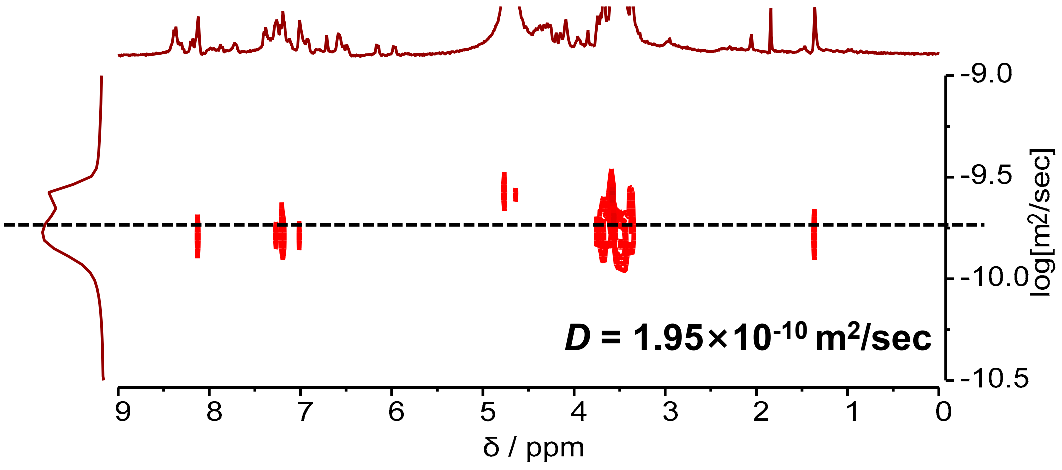

Figure S14. DOSY spectrum of **bis-TO@(*S*)-C[4]B** (298 K, 2.0×10^{-3} M in D_2O).

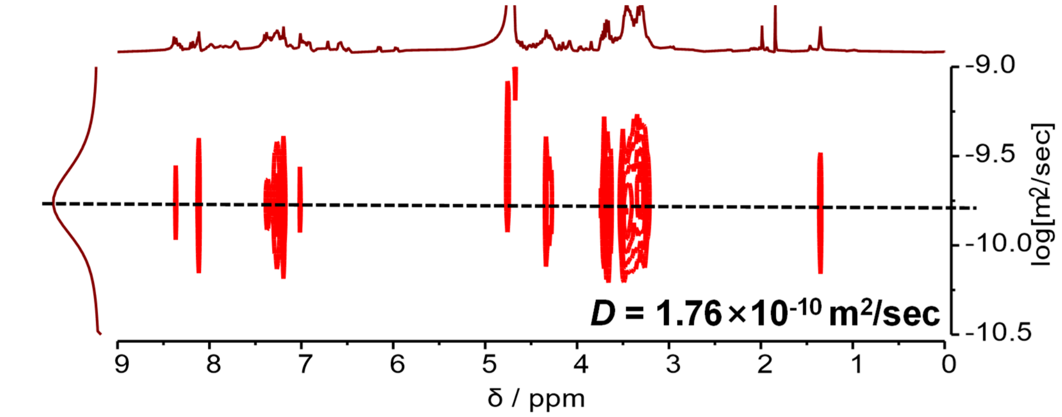

Figure S15. DOSY spectrum of **bis-TO@(*S*)-C[4]B** (298 K, 3.0×10^{-3} M in D_2O).

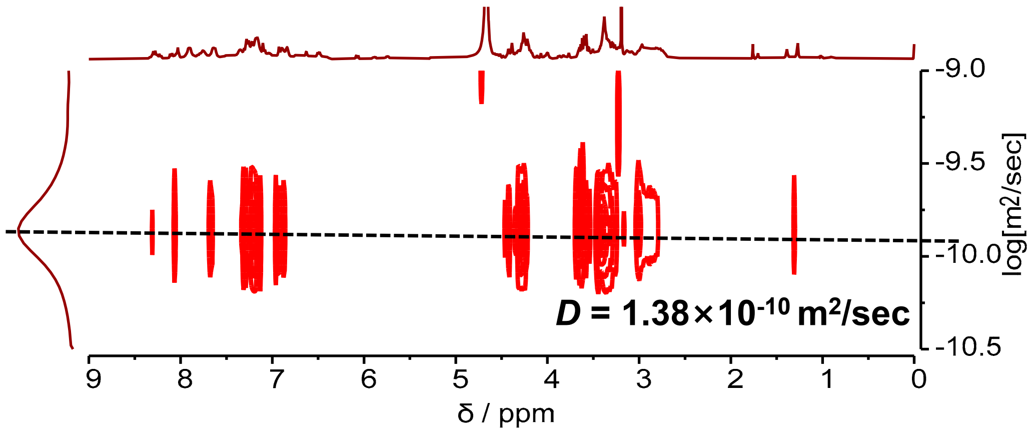

Figure S16. DOSY spectrum of **bis-TO@(*S*)-C[4]B** (298 K, 5.0×10^{-3} M in D_2O).

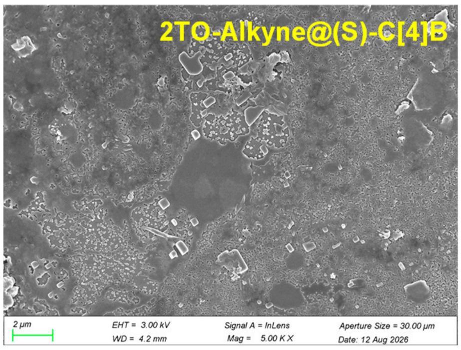

Figure S17. SEM images of **2TO-Alkyne@(*S*)-C[4]B** at concentration of 10.0 μM.

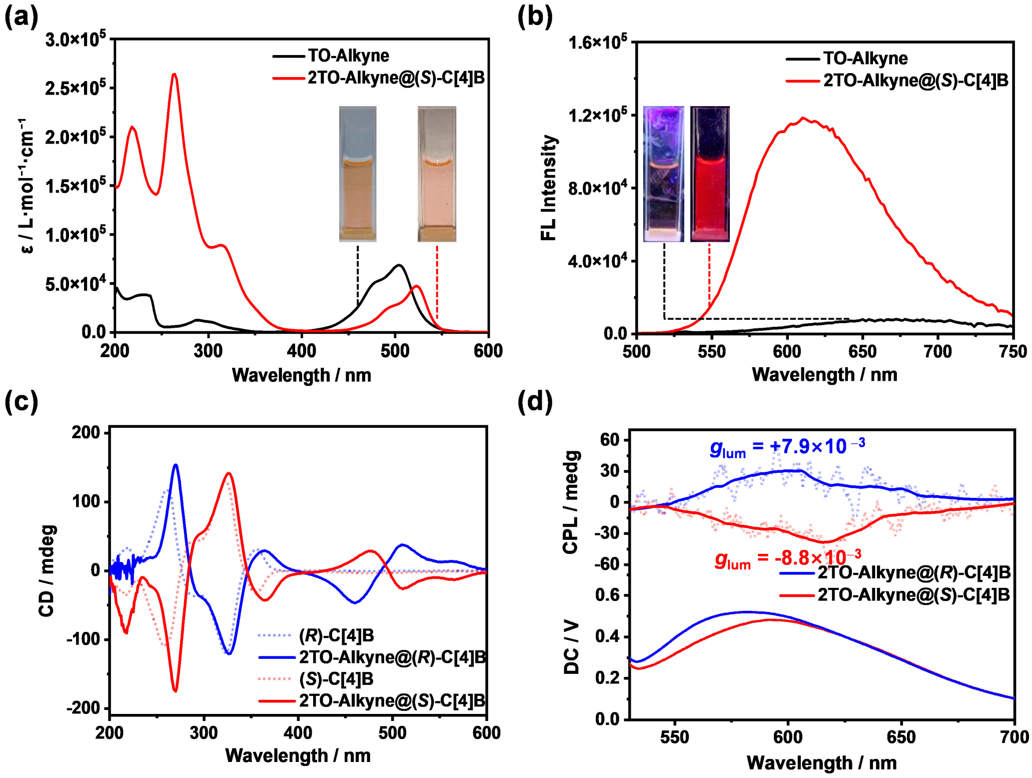

Figure S18. (a) UV-Vis spectra, (b) Fluorescence spectra (H_2O, 10.0 μM, 298 K) of **TO-Alkyne**, 2**TO-Alkyne@(*S*)-C[4]B**. (c) CD spectra of (*R*)-/(*S*)**-C[4]B**, 2**TO-Alkyne@**(*R*)-/(*S*)**-C[4]B**. (d) CPL spectra (H_2O, 10.0 μM, 298 K) of 2**TO-Alkyne@**(*R*)-/(*S*)**-C[4]B**.

Table S1. The g_{lum} values for CPL emission of aqueous chiral supramolecular assemblies.

System	$|g_{lum}|$	Reference
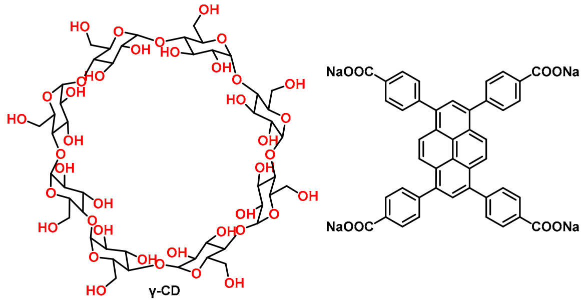 γ-CD	3.27×10^{-3} (λ=460 nm)	[50]
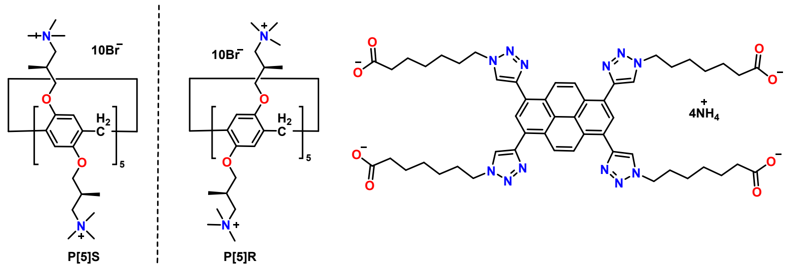 P[5]S, P[5]R	1.7×10^{-3} (λ=550 nm)	[51]
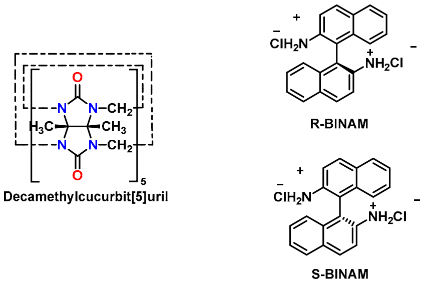 Decamethylcucurbit[5]uril, R-BINAM, S-BINAM	1.17×10^{-3} (λ=400 nm)<br>1.46×10^{-3} (λ=400 nm)	[52]
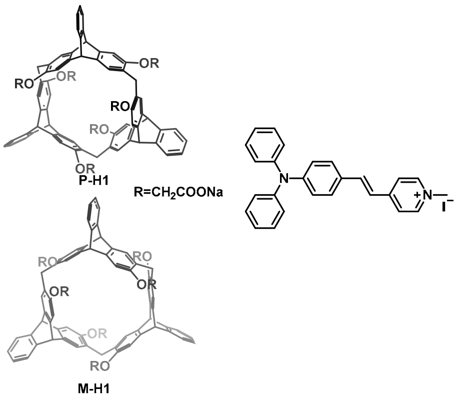 P-H1, M-H1, R=CH_2COONa	2.67×10^{-4} (λ=655 nm)<br>1.48×10^{-4} (λ=655 nm)	[53]
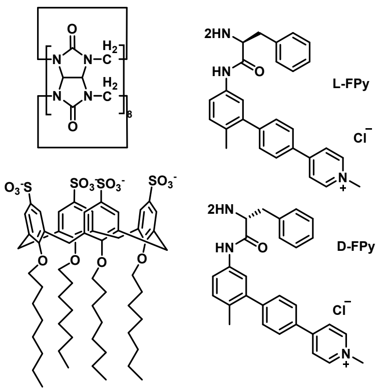 L-FPy, D-FPy	1.7×10^{-4} (λ=350 nm)<br>1.07×10^{-4} (λ=350 nm)	[54]

System	$\lvert g_{lum} \rvert$	Reference
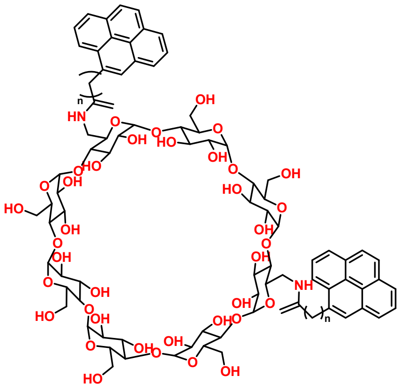	5.3×10^{-2} (λ=550 nm)	[55]
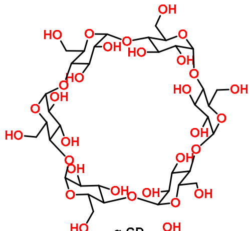 α-CD 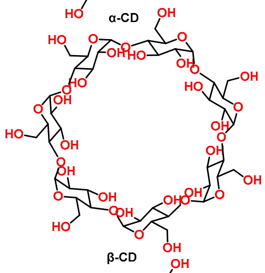 β-CD 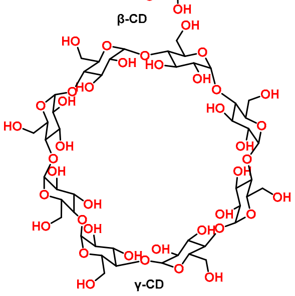 γ-CD 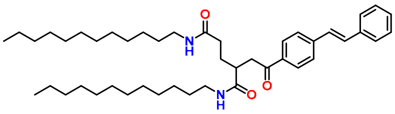	1.24×10^{-2} (λ=390 nm)<br>9.0×10^{-3} (λ=390 nm)<br>1.92×10^{-2} (λ=390 nm)	[56]
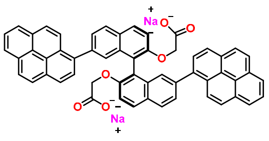 R-B-Pry 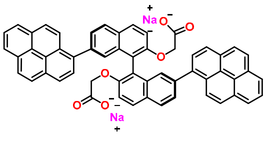 S-B-Pry 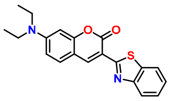 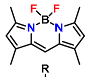 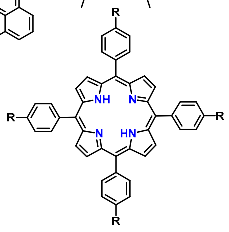 R=COOH	9.0×10^{-3} (λ=510 nm)<br>1.2×10^{-4} (λ=520 nm)<br>1.1×10^{-3} (λ=680 nm)	[57]

System	$\lvert g_{lum}\rvert$	Reference
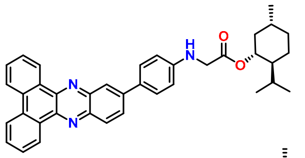	6.28×10^{-3} (λ=460 nm)	[58]
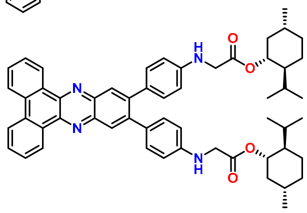	3.01×10^{-2} (λ=460 nm)	
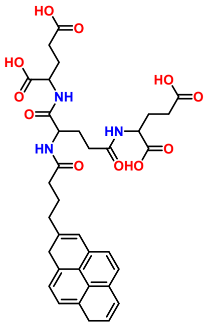	6.87×10^{-3} (λ=475 nm)	[59]
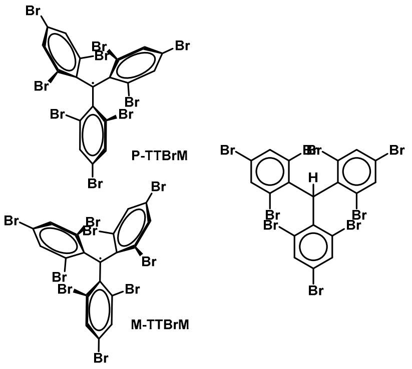	0.65×10^{-3} (λ=595 nm)<br>0.4×10^{-3} (λ=720 nm)	[60]
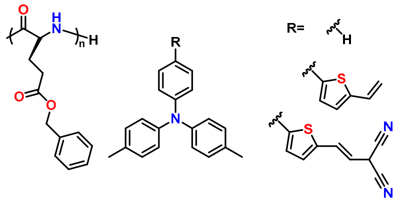	0.5×10^{-2} (λ=370 nm)<br>0.7×10^{-2} (λ=525 nm)<br>1.1×10^{-2} (λ=648 nm)	[61]
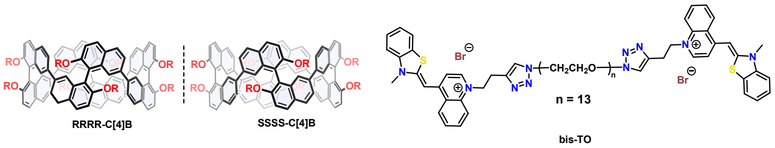	8.5×10^{-3} (λ=625 nm)	This work

50 Jin, S.; Qi, S.; Yang, L.-P.; Xu, Z.; Yang, L.; Cao, X.; Wu, G.; Sun, Y.; Jin, S.; Qi, S.; Yang, L.-P.; Xu, Z.; Yang, L.; Cao, X.; Wu, G.; Sun, Y. γ-Cyclodextrin-Based 2:2 Host–Guest Complexes With Emergent Circularly Polarized Luminescence. *Aggregate* **2026**, *7*, e70375.

51 Fa, S.; Tomita, T.; Wada, K.; Yasuhara, K.; Ohtani, S.; Kato, K.; Gon, M.; Tanaka, K.; Kakuta, T.; Yamagishi, T.-a.*; et al.*; Fa, S.; Tomita, T.; Wada, K.; Yasuhara, K.; Ohtani, S.; Kato, K.; Gon, M.; Tanaka, K.; Kakuta, T.; Yamagishi, T.-a.*; et al.* CPL on/off control of an assembled system by water soluble macrocyclic chiral sources with planar chirality. *Chemical Science* **2022**, *13*, 5846-5853.

52 An, B.; Ge, Q.; Cong, H.; Gao, R.; An, B.; Ge, Q.; Cong, H.; Gao, R. Circularly polarized luminescence from

decamethylcucurbit[5]uril-triggered chiral supramolecular hydrogels. *Chemical Communications* **2025**, *61*, 7478-7481.

53 Guo, Y.; Han, Y.; Chen, C.-F.; Guo, Y.; Han, Y.; Chen, C.-F. Construction of Chiral Nanoassemblies Based on Host-Guest Complexes and Their Responsive CD and CPL Properties: Chirality Transfer From 2,6-helic[6]arenes to a Stilbazolium Derivative. *Frontiers in Chemistry* **2019**, *Volume 7 - 2019*,

54 Li, Y.; Wu, J.; Zhang, W.; Zhang, H.; Zhou, W.-L.; Liu, Y.; Li, Y.; Wu, J.; Zhang, W.; Zhang, H.; Zhou, W.-L.; Liu, Y. Cucurbit[8]Uril Achieved Single-Molecule Chiral Transfer to Supramolecular Circularly Polarized Luminescence. *Advanced Science* **2026**, *13*, e75360.

55 Tu, C.; Wu, W.; Liang, W.; Zhang, D.; Xu, W.; Wan, S.; Lu, W.; Yang, C.; Tu, C.; Wu, W.; Liang, W.; Zhang, D.; Xu, W.; Wan, S.; Lu, W.; Yang, C. Host–Guest Complexation-Induced Aggregation Based on Pyrene-Modified Cyclodextrins for Improved Electronic Circular Dichroism and Circularly Polarized Luminescence. *Angewandte Chemie International Edition* **2022**, *61*, e202203541.

56 Yang, C.; Yang, D.; Zhu, X.; Meng, Y.; Liu, M.; Yang, C.; Yang, D.; Zhu, X.; Meng, Y.; Liu, M. Circularly Polarized Luminescence of Langmuir–Schaefer Films of Amphiphilic Stilbene Enhanced via Interfacial Reaction with Cyclodextrins. *Langmuir* **2020**, *36*, 12366-12374.

57 Zhang, Q.; Song, Z.; Ma, Y.; Guo, D.-S.; Cai, K.; Zhang, Q.; Song, Z.; Ma, Y.; Guo, D.-S.; Cai, K. Chiral Micellar Assemblies for Circularly Polarized Luminescence Amplification, Dye Encapsulation, and Multilevel Information Encryption. *Angewandte Chemie International Edition* **2026**, *65*, e4171255.

58 Kaur, N.; Labella, J.; Seki, S.; Kumar, M.; Bhalla, V.; Kaur, N.; Labella, J.; Seki, S.; Kumar, M.; Bhalla, V. Regulating the Morphology-Dependent Chiroptical Properties and Photoconductivity in Supramolecularly Orchestrated Phenazine-Based Assemblies in Aqueous Media. *ACS Applied Materials & Interfaces* **2026**, *18*, 27030-27042.

59 Zhang, Y.; Yang, D.; Han, J.; Zhou, J.; Jin, Q.; Liu, M.; Duan, P.; Zhang, Y.; Yang, D.; Han, J.; Zhou, J.; Jin, Q.; Liu, M.; Duan, P. Circularly Polarized Luminescence from a Pyrene-Cyclodextrin Supra-Dendron. *Langmuir* **2018**, *34*, 5821-5830.

60 Schievano, G.; Gonzalez-Pato, N.; Míguez-Lago, S.; Armada, P.; Cerdá, J.; Aragó, J.; Veciana, J.; Campaña, A. G.; Ratera, I.; Mayorga-Burrezo, P.; Schievano, G.; Gonzalez-Pato, N.; Míguez-Lago, S.; Armada, P.; Cerdá, J.; Aragó, J.; Veciana, J.; Campaña, A. G.; Ratera, I.; Mayorga-Burrezo, P. Intrinsically Chiral Excimers: Water-Compatible Trityl-Based Nanoparticles as Tailored Dual Emitters of Circularly Polarized Luminescence in the Vis or NIR Regions. *Small* **2026**, *22*, e12627.

61 Ye, Y.; Hu, X.; Yang, D.; Gu, Q.; Liu, S.; Jiang, J.; Liu, G.; Du, J.; Ye, Y.; Hu, X.; Yang, D.; Gu, Q.; Liu, S.; Jiang, J.; Liu, G.; Du, J. Aqueously dispersed homopolypeptide nanotoroids with tunable circularly polarized luminescence. *Chinese Chemical Letters* **2026**, *37*, 111844.
